# Extracellular Vesicle-Associated microRNAs as Candidate Biomarkers and Mediators of Diabetic Complications: Clinical and Translational Evidence Across Neuropathy, Diabetic Kidney Disease, Retinopathy, and MASLD

**DOI:** 10.3390/metabo16070500

**Published:** 2026-07-16

**Authors:** Raúl Ibarra-Salce, José Luis Eduardo Doval-Caballero, Daniel Uribe-Cortés, Genesis Dinora Eugenio-Ponce, Mariela Ibarra-Salce, Omar Jaime-Leal, Manuel Ramón García-Sáenz

**Affiliations:** 1 Facultad de Medicina Unidad Saltillo, Universidad Autónoma de Coahuila, Saltillo 25160, Mexico; raul_ibarra@uadec.edu.mx; 2 Clínica de Obesidad, Instituto Nacional de Ciencias Médicas y Nutrición “Salvador Zubirán”, Mexico City 14080, Mexico; eduardo.dovalc@incmnsz.mx; 3 Hospital Ángeles Clinica Londres, Mexico City 06700, Mexico; daniel.uribec@endocrino.mx; 4 Hospital San Angel Inn Satelite, Naucalpan de Juárez 53100, Mexico; genesis.eugeniop@gmail.com; 5 Servicio de Nefrología, Hospital General de Zona N. 2, Saltillo 25240, Mexico; mariela.ibarra.s@gmail.com; 6 Intus Digestiva, Hospital Ángeles Torreón, Torreón 27250, Mexico; dr.omarjaime.gastro@gmail.com; 7 Servicio de Endocrinología, Hospital de Especialidades “Dr. Bernardo Sepúlveda” Centro Médico Nacional Siglo XXI, Instituto Mexicano del Seguro Social, Avenida Cuauhtémoc 330, Col. Doctores, Del. Cuauhtémoc, Mexico City 06720, Mexico

**Keywords:** type 2 diabetes, extracellular vesicles, microRNAs, diabetic neuropathy, diabetic kidney disease, diabetic retinopathy, MASLD, biomarkers

## Abstract

Background/Objectives: Type 2 diabetes is increasingly recognized as a systemic disorder driven not only by chronic hyperglycemia and insulin resistance, but also by dysregulated interorgan communication. Extracellular vesicles (EVs), including exosomes and microvesicles, have emerged as biologically active carriers of proteins, lipids, and microRNAs capable of modulating gene expression in recipient cells. This narrative review integrates clinical, experimental, and translational evidence on EV-associated microRNAs as candidate biomarkers and potential mediators of diabetic complications, with emphasis on diabetic neuropathy, diabetic kidney disease, diabetic retinopathy, and metabolic dysfunction-associated steatotic liver disease (MASLD). Methods: This review was aligned with the SANRA framework and focused on biological plausibility, evidence from tissue and biofluids, biomarker potential, therapeutic implications, and barriers to clinical translation. Studies were additionally interpreted according to biological matrix, EV-carrier specificity, analytical platform, study design, and level of functional validation. Results: Across complications, EV-associated microRNAs appear to participate in shared pathogenic processes, including oxidative stress, inflammation, endothelial dysfunction, fibrosis, angiogenesis, neurodegeneration, and metabolic memory. In diabetic neuropathy, microRNAs such as miR-146a, miR-155, miR-21-5p, and miR-148a-3p have been linked to neuroinflammation, Schwann-cell dysfunction, axonal injury, and neuropathic pain. In diabetic kidney disease, miR-21, miR-29, miR-30, and miR-126 are implicated in podocyte injury, tubulointerstitial fibrosis, albuminuria, and microvascular dysfunction. In diabetic retinopathy, microRNAs including miR-146a, miR-155, miR-21, miR-126, and miR-200b contribute to neurovascular injury, inflammation, barrier disruption, and angiogenesis. In MASLD associated with diabetes, hepatocyte-derived EVs carrying microRNAs such as miR-1 and miR-126a-3p may link hepatic lipotoxicity to endothelial inflammatory and β-cell dysfunction. Conclusions: Although EV-associated microRNAs offer promising opportunities for biomarker discovery, risk stratification, and targeted therapies, clinical translation remains limited by heterogeneity in EV isolation, microRNA quantification, biological matrices, and outcome definitions. Distinguishing EV-associated miRNAs from total circulating extracellular miRNAs remains essential for biological interpretation. Standardized, longitudinal, and externally validated studies are required before these signals can be implemented as actionable tools in precision diabetes care.

## 1. Introduction

Type 2 diabetes (T2D) is a systemic metabolic disease characterized by insulin resistance, chronic low-grade inflammation, and progressive pancreatic β-cell dysfunction. Although traditionally interpreted as the sum of independent tissue alterations, it is now recognized that its progression and the development of micro- and macrovascular complications are due to dysregulated interorgan communication networks [1,2].

T2D should therefore be framed as a complex systemic disorder rather than as an isolated disturbance of glucose homeostasis. Beyond hyperglycemia and insulin resistance, its progression involves interacting networks of low-grade inflammation, oxidative stress, mitochondrial dysfunction, endothelial injury, adipose tissue remodeling, hepatic steatosis, immune activation, gut barrier dysfunction, microbiota-derived signals, neural vulnerability, and even experimental evidence also supports the relevance of extra-pancreatic therapeutic axes. For example, modulation of the gut microbiota–mucosal barrier axis by Astragalus membranaceus polysaccharides improved glycolipid metabolism, inflammation, oxidative stress, organ injury, and intestinal integrity in T2D mice [3]. In parallel, integrated transcriptomic and metabolomic analyses of insulin-based interventions have shown that insulin-related signaling can influence neuroinflammation, neuronal apoptosis, axon guidance, synaptic pathways, and metabolic networks in experimental neurovascular disease [4]. These findings support a network-based interpretation in which diabetic complications emerge from disrupted interorgan communication and tissue-specific susceptibility rather than from hyperglycemia alone [3,4].

In this context, extracellular vesicles (EVs), including exosomes and microvesicles, as defined by the original studies, have emerged as potential carriers of systemic signaling [5,6]. More than just cellular byproducts, EVs are biological nanoparticles capable of transporting proteins, lipids, and genetic material, including microRNAs, to distant recipient cells, where they can modulate gene expression networks and cellular phenotypes [5,7,8,9,10]. Therefore, EVs are currently considered active components of physiological homeostasis and the propagation of pathological processes [9,10,11,12].

The accumulating evidence in cardiometabolic disease suggests that EVs not only act as circulating biomarkers but also as active bioeffectors in inflammation, atherogenesis, endothelial dysfunction, and the progression of T2D [13,14,15,16,17]. This potential dual role as biomarker and functional mediator is particularly relevant for diabetic complications, although the strength of evidence varies substantially across organs, biological matrices, and experimental designs. This narrative review integrates clinical and translational evidence on microRNAs (miRNAs) associated with EVs in diabetic neuropathy, diabetic kidney disease, diabetic retinopathy, and diabetes-related MASLD, with an emphasis on their role as biomarkers, pathophysiological mediators, and potential therapeutic targets.

## 2. Materials and Methods

We conducted a narrative review with a clinical and translational focus to integrate the available evidence on miRNAs associated with microvesicles and EVs as mediators of complications in patients with diabetes. This manuscript was structured around six thematic axes: interorgan communication in diabetes, diabetic neuropathy, diabetic kidney disease, diabetic retinopathy, diabetes-associated MASLD, and clinical/translational perspectives. This review was aligned with the SANRA (Scale for the Assessment of Narrative Review Articles) domains, including justification of the topic, explicit objectives, literature search, referencing, scientific reasoning, and appropriate data presentation [18].

The literature search was designed to identify mechanistic, preclinical, clinical, and translational literature. Priority was given to original studies in humans or experimental models measuring miRNAs associated with extracellular vesicles, exosomes, or microvesicles; studies linking miRNAs to functional, histological, or clinical outcomes; and recent reviews useful for contextualizing mechanisms. Articles in English or Spanish were considered, without applying strict design restrictions, due to the narrative nature of this manuscript.

The search was performed in PubMed using combinations of terms related to diabetes, extracellular vesicles, miRNAs, and diabetes complications. The main concepts included: “Diabetes Mellitus”, “diabet*”, “Extracellular Vesicles”, “Exosomes”, “Microvesicle”, “ectosome”, “microRNAs”, “microRNA”, “miRNA”, “cell communication”, “intercellular communication”, “epigenetic”, “endothelial dysfunction”, “diabetic neuropathies”, “diabetic neuropath*”, “peripheral neuropath*”, “neuropathic pain”, “diabetic nephropathies”, “diabetic kidney disease”, “diabetic nephropath*”, “albuminuria”, “glomerulosclerosis”, “podocyte”, “diabetic retinopathy”, “diabetic retinopath*”, “proliferative diabetic retinopathy”, “diabetic macular edema”, “retinal neurodegeneration”, “metabolic dysfunction-associated steatotic liver disease”, “MASLD”, “MASH”, “Non-alcoholic fatty liver disease”, “NAFLD”, and “fatty liver”.

The synthesis was organized by diabetic complication and by level of evidence: biological plausibility, experimental evidence, evidence in biofluids or human tissue, potential utility as biomarkers, and translational barriers. Since many studies use the terms “exosomes,” “microvesicles,” and “EVs” interchangeably, this review used “EVs” as a general term when vesicular biogenesis was not specifically demonstrated and retained the terms “exosomes” or “microvesicles” when so defined by the original authors. Terminology was additionally aligned with the Minimal Information for Studies of Extracellular Vesicles 2023 (MISEV2023) recommendations, emphasizing that particle size, density, surface markers, and isolation method alone are insufficient to prove EV subtype biogenesis [19].

### Evidence Stratification and Methodological Appraisal

For this revision, the evidence was additionally stratified according to: (i) biological matrix or sample type, including serum, plasma, whole blood, urine, vitreous humor, tissue, or cell-culture supernatant; (ii) carrier specificity, distinguishing EV-associated miRNAs from total circulating miRNAs when possible; (iii) EV isolation or enrichment method, including ultracentrifugation, size-exclusion chromatography, precipitation-based kits, immunocapture, or mixed approaches; (iv) EV characterization, including particle size, concentration, morphology, surface or luminal markers, and assessment of non-vesicular contaminants; (v) miRNA analytical platform, including qPCR, small RNA sequencing, or targeted panels; (vi) normalization strategy; (vii) study design, including preclinical, cross-sectional clinical, case–control, longitudinal, or interventional studies; and (viii) clinical outcome definition.

Because the included studies differed substantially in sample source, EV isolation, miRNA quantification, normalization, comparator groups, and clinical outcome definitions, the synthesis emphasizes biological plausibility, reproducibility across matrices, level of functional validation, and translational readiness.

A methodological stratification table was added as Supplementary Table S1 to summarize biological matrix, EV-carrier specificity, EV isolation or enrichment approach, EV characterization or reporting, miRNA analytical platform and normalization approach when extractable, study design, sample size or validation status, clinical outcome definition, evidence level, and the main limitation of each evidence group. 

## 3. MicroRNAs in Extracellular Vesicles as an Interorgan Communication System in Diabetes

Consistent with the MISEV2023 recommendations, the term “extracellular vesicle” is used in this review as an operational descriptor for lipid bilayer-delimited particles released by cells, particularly when vesicle biogenesis has not been experimentally demonstrated. The terms “exosome” and “microvesicle” are retained only when used by the original authors and when the study provides sufficient evidence regarding vesicle enrichment, size range, markers, or biogenesis. This distinction is important because particle size, density, surface markers, and isolation method alone are insufficient to prove endosomal or plasma-membrane origin. Therefore, functional effects attributed to “exosomes” in older or incompletely characterized studies should be interpreted as effects of EV-enriched preparations unless vesicle subtype identity was rigorously established [19].

EVs constitute a heterogeneous family of particles delimited by a lipid bilayer and released by virtually all cell types. They include exosomes, derived from the endosomal pathway; microvesicles or ectosomes, originated from direct budding from the plasma membrane, and apoptotic bodies. However, these terms should be interpreted cautiously unless vesicle biogenesis has been specifically demonstrated. Given the overlap in size, markers, and isolation methods, the general term EVs is more appropriate when there is no comprehensive characterization of vesicular biogenesis, while “exosomes” or “microvesicles” should be reserved for studies that clearly document their origin or specific enrichment [2,5,6,7,8,9,10,11,12].

In type 2 diabetes, insulin resistance, and states of metabolic inflammation, an increase in circulating EVs derived from platelets, leukocytes, endothelium, and metabolically active tissues has been described. These EVs do not act solely as cellular “debris”, rather they transport proteins, lipids, mRNA, and miRNA protected by the lipid bilayer, which facilitates their stability in plasma, urine, and other biofluids [11,13,14,15,16,17,20,21,22,23].

EV-associated miRNAs may function as biological effectors when vesicle release, recipient-cell uptake, miRNA transfer, target repression, and phenotypic effects are demonstrated. Experimental evidence indicates that EVs derived from obese adipocytes can influence insulin signaling in hepatocytes and myocytes, whereas circulating Evs from insulin-resistant individuals can transcriptomically reprogram monocytes [14,22,24]. However, clinical detection of an EV-associated miRNA should be interpreted as biomarker-level or hypothesis-generating evidence unless functional vesicular transfer has been demonstrated.

The selection of miRNAs within EVs does not appear to be a passive reflection of the cytoplasmic transcriptome, but rather a process regulated by vesicular biogenesis machinery and RNA-binding proteins, such as hnRNPA2B1, YBX1, Ago2, and SYNCRIP. Chronic hyperglycemia, oxidative stress, NF-κβ activation, mitochondrial dysfunction, and low-grade inflammation modify both the number and content of Evs, favoring a pro-inflammatory or diabetogenic miRNA load. This mechanism supports the concept of diabetes as a disease of altered interorgan communication, in which adipose tissue, liver, endothelium, kidney, retina, peripheral nerve, and pancreatic islets exchange signals that can amplify local and systemic damage [5,6,7,8,9,10,11,12].

Compared with non-vesicular extracellular miRNAs, including miRNAs bound to Argonaute proteins or associated with lipoproteins, vesicular packaging confers protection against Rnase degradation, that may facilitate tissue targeting through surface molecules, and allows for integration with other molecular cargoes. This explains their appeal as biomarkers and, at the same time, highlights the fact that interpreting a circulating miRNA without knowing its carrier can lead to incomplete conclusions [14,22,24].

### 3.1. Biogenesis, Heterogeneity, and Operational Nomenclature of EVs

The incorporation of miRNAs into EVs is an active and regulated process that depends on the cellular metabolic state and vesicular biogenesis machinery [7,10,12]. Exosomes are generated primarily from the formation of intraluminal vesicles in multivesicular bodies (MVBs), through mechanisms dependent on the ESCRT system (ESCRT-0, -I, -II, -III), along with accessory proteins such as TSG101, ALIX, and VPS4. However, there are also ESCRT-independent pathways, regulated by ceramide and tetraspanins (CD63, CD81, CD9), which allow formation of specific microdomains for cargo selection [7].

Heterogeneity in vesicular biogenesis and composition has been extensively documented [10,12]. Therefore, the microRNA content in EVs should be interpreted as the result of a selective packaging process, influenced by RNA-binding proteins, post-transcriptional modifications, and intracellular stress signals, rather than as a simple copy of the cellular transcriptome.

Accordingly, evidence of EV-mediated miRNA function should not rely solely on differential miRNA abundance in an EV-enriched fraction. Stronger mechanistic evidence requires demonstration of vesicle release by a defined donor cell, uptake by a biologically relevant recipient cell, transfer of miRNA cargo, repression of a validated target, reproduction of the phenotype by miRNA gain-of-function, attenuation by miRNA inhibition, and ideally rescue experiments. Without these elements, EV-associated miRNAs should be interpreted primarily as biomarkers or candidate mediators rather than as proven causal effectors.

Under conditions of insulin resistance, significant changes in the protein profile of circulating EVs have been described, especially those internalized by leukocytes [14]. Additionally, EVs have been implicated in the modulation of metabolic inflammation and the progression of type 2 diabetes [15,16]. These findings suggest that chronic hyperglycemia not only alters intracellular signaling but also reprograms the exported vesicular contents, favoring the selective externalization of pro-inflammatory and diabetogenic microRNAs [5,6,7,8,9,10,11,12].

In models of obesity and insulin resistance, EVs derived from obese adipocytes contain miRNAs capable of inducing insulin resistance in hepatocytes and myocytes [24]. Furthermore, EVs internalized by monocytes can activate oxidative stress and inflammatory pathways, amplifying the low-grade chronic inflammation characteristic of type 2 diabetes [13,14]. At the cardiovascular level, EVs participate in angiogenesis, vascular inflammation, and atherogenesis [23], while at the renal level, urinary EVs constitute a potential source of biomarkers in diabetic kidney disease and associated hypertension [25].

**Figure 1:**
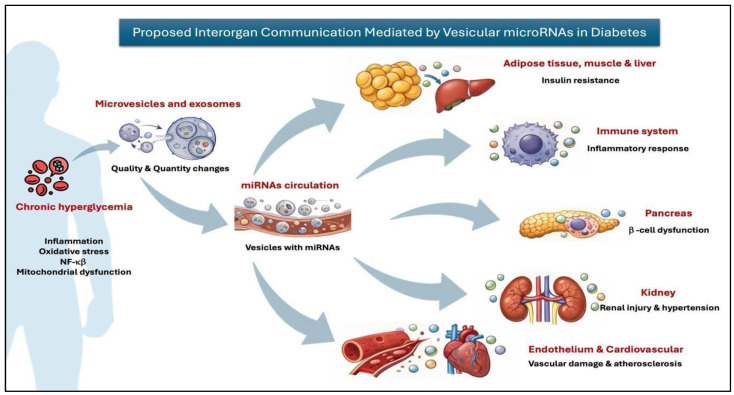
Model of vesicular miRNA-mediated interorgan communication in diabetes. Chronic hyperglycemia induces quantitative and qualitative changes in microvesicles and exosomes, modifies vesicular miRNA sorting, and promotes the systemic circulation of proinflammatory signals. When vesicle subtype biogenesis is not experimentally demonstrated, these particles should be interpreted operationally as EVs or EV-enriched preparations. These EV-associated miRNAs can be internalized by metabolic and immune cells and have been linked to insulin resistance, pancreatic β-cell dysfunction, kidney damage, vascular inflammation, and atherogenesis in experimental and translational studies. The figure summarizes the central concept of this review: EV-associated miRNAs may reflect metabolic injury and, when functional transfer is demonstrated, may participate in interorgan communication involved in diabetic complications.

Taken together, this model suggests that hyperglycemia drives EV reprogramming inducing release of proinflammatory miRNAs, their systemic dissemination, and uptake by metabolic and immune tissues. This establishes a pathologic circuit that amplifies insulin resistance, inflammation, and multisystem damage. Figure 1 summarizes the conceptual framework by integrating biogenesis, systemic circulation of miRNAs, and their general impact on different tissues.

### 3.2. Implications for Biomarkers: Carrier, Stability, and Biological Significance

The advantage of studying miRNAs associated with EVs extends beyond their detectability. These molecules provide biological information about the tissue of origin and the functional state of the donor cell. In diabetes, a given miRNA could circulate through different carriers, such as Argonaute proteins, lipoproteins, or can be encapsulated in vesicles, which may lead to different biodistribution patterns and biological functions [14,22,24].

A further distinction is required between EV-associated miRNAs and total extracellular miRNAs. Extracellular miRNAs can be encapsulated within EVs, bound to Argonaute proteins, associated with high- or low-density lipoproteins, or be present in other ribonucleoprotein complexes. These carriers differ in stability, biodistribution, cellular uptake, and potential biological activity. Therefore, clinical associations based on total serum or plasma miRNAs should not be interpreted as evidence of EV-mediated communication unless the vesicular carrier has been isolated, characterized, and functionally tested.

Therefore, vesicular microRNA studies should explicitly report the biological matrix, the vesicular isolation method, purity controls, the normalization strategy, the analytical platform, and the relationship with longitudinal clinical outcomes. Whenever possible, studies should also report whether the analyzed miRNAs are EV-associated, total circulating, Argonaute-bound, lipoprotein-associated, or present in mixed extracellular RNA fractions. This standardization is essential to distinguish whether a microRNA signature represents a biomarker of metabolic status, a functional mediator of tissue damage, or both.

The lipid bilayer of EVs protects miRNAs from degradation by RNases and oxidative stress [11]. Although Ago2-associated miRNAs also exhibit stability, EV-associated and non-vesicular extracellular miRNAs may differ in biodistribution, cellular uptake, and biological interpretation [14,22,24]. Therefore, subsequent sections distinguish EV-associated evidence from total circulating miRNA evidence whenever possible.

## 4. Clinical and Translational Evidence of EV-Associated and Circulating miRNAs in Diabetic Neuropathy

Diabetic neuropathy (DN) is a heterogeneous complication that combines axonal degeneration, Schwann-cell dysfunction, neuroinflammation, oxidative stress, endothelial alterations, and, in a subset of patients, neuropathic pain [26,27]. miRNAs should not be interpreted as a sole cause of DN, but rather as post-transcriptional regulators capable of modulating the intensity, timing, and direction of neural and glial damage.

### 4.1. MicroRNA Mechanisms in Neuroinflammation, Oxidative Stress, and Glial Dysfunction

One of the best-characterized circuits in experimental models is that of miR-146a. Chronic hyperglycemia reduces miR-146a in dorsal root ganglion neurons, releasing IRAK/TRAF6 signaling and promoting pro-inflammatory responses; experimental restoration of miR-146a partially reverses neuronal impairment [28]. Complementarily, miR-155 has been linked to NF-κβ-dependent inflammatory pathways, while other miRNAs, such as miR-9, participate in axes related to neuronal excitability and pain [26,27]. These findings support the idea that neuroinflammation in DN does not depend on a single miRNA, but on an imbalance of regulatory networks that affect neurons, glial cells, and peripheral immunity.

Oxidative stress constitutes another central axis. In db/db models, miR-25 is reduced while ROS and Nox4 are increased; experimental restoration of miR-25 reduces PKC and Nox4 activation and attenuates the AGE-RAGE pathway, supporting a protective role [29]. Similarly, in the dorsal root ganglion, miR-106a is reduced in DN, and its experimental overexpression reverses hyperglycemia-induced effects by downregulating 12/15-LOX, a mediator of oxidative stress [30]. Taken together, these data suggest that the loss of antioxidant miRNAs can amplify neuronal damage, although the direction of the effect depends on the miRNA, cell type, and experimental model.

Schwann cell dysfunction constitutes a particularly relevant bridge between molecular biology and clinical phenotype. Under physiological conditions, these cells preserve neuronal structure and function, and coordinate remyelination after injury; however, if their adaptive responses fail, they may amplify axonal degeneration. In adult peripheral nerves, several injury-regulated microRNAs inhibit Schwann cell dedifferentiation/proliferation factors, facilitating the transition to pro-myelinating states; for example, miR-34a can suppress Notch1 and Ccnd1, while miR-140 targets Egr2, a master regulator of myelination [31]. Therefore, miRNAs should be considered regulators of the glial repair program, not merely passive markers of neuronal damage.

Donor cell-specific EV communication is particularly relevant in this context, as Schwann cell-derived EVs may exert beneficial or deleterious effects depending on the metabolic state of the donor cell. Exosomes derived from high-glucose-stimulated Schwann cells were reported to be enriched in miR-28, miR-31a, and miR-130a, which reduced axonal growth in dorsal root ganglion neurons, promoting features of diabetic peripheral neuropathy in db/db mice, including impaired nerve conduction and reduced intraepidermal nerve fiber density [32]. This finding indicates that Schwann cells are not only passive targets of diabetic injury but may also export pathogenic vesicular cargo capable of altering axonal integrity. Conversely, exosomes derived from Schwann cells under reparative experimental conditions have been associated with remyelination, axonal support, and functional improvement [33,34]. These apparently divergent findings reinforce the need to define donor cell status, culture conditions, EV cargo, and recipient-cell effects before assigning a uniformly protective or pathogenic role to EVs.

In human tissue, miR-21-5p has emerged as a relevant candidate signal, although current evidence remains limited. Small RNA analysis of human sural nerves from patients with DN identified extensive microRNA dysregulation, with miR-21-5p emerging as a prominent signal localized to SOX10+ Schwann cells and associated with axonal loss. The integrated miRNA-mRNA analysis showed a negative correlation with targets such as DOK3, PLD1, and MAPK10, with enrichment in axonal guidance pathways, MAPK/Ras signaling, and neurotrophic response [35]. The localization of miR-21-5p in Schwann cells and its association with axonal loss support its potential relevance for glial injury and repair failure, but this evidence should not be interpreted as proof of EV-mediated causality. However, because this evidence is currently derived from a preprint and tissue-based observational analysis, it should be interpreted as preliminary human tissue evidence rather than definitive proof of EV-mediated causality.

The functional relevance of microRNAs in Schwann cells is also supported by conditional Dicer deletion models. In PLP-cKO mice, loss of microRNA processing in Schwann cells induces peripheral neuropathy by reducing promyelinating miRNAs, and increasing inhibitory regulators such as c-Jun, Notch, and Sox2, and PTEN. These changes disrupt Schwann cell differentiation, myelination, and axonal support. Notably, treatment with Schwann cell-derived exosomes (SC-Exo) reverses several of these alterations [27,33]. This model provides functional evidence that microRNA-mediated post-transcriptional regulation is directly involved in glial homeostasis and nerve repair capacity. Nevertheless, as with other EV studies, interpretation depends on the extent of vesicle characterization, isolation purity, cargo attribution, and demonstration that the observed effects are mediated by vesicular miRNAs rather than by co-isolated proteins, lipoproteins, or soluble factors.

Neuropathic pain represents a particularly relevant clinical dimension. Genomic screening identified miR-33 and miR-380 as factors involved in neuropathic pain, and miR-124-1 as a mediator of physiological nociception [36]. Furthermore, miR-34a-5p has been reported to be elevated in patients with diabetic neuropathic pain, with discriminative capacity for diabetic neuropathy and a positive correlation with triglycerides, fasting glucose, and glycated hemoglobin [37]. These data suggest that selected miRNAs may help characterize painful, inflammatory, or axonal-injury phenotypes in future studies, but current evidence remains primarily associative [36,37]. However, most pain-related miRNA studies remain mechanistic or associative and should not be interpreted as EV-mediated effect, unless vesicular localization and functional transfer are directly demonstrated.

### 4.2. Circulating Biomarkers and Diagnostic Panels

The diagnostic utility of circulating miRNAs is based on two properties: their stability in biological fluids and the possibility that their dysregulation may precede established clinical manifestations [27,38]. However, the current landscape of biomarkers in DN is heterogeneous, with relevant differences between studies according to sample type, analytical platform, definition of neuropathy, sample size and normalization strategy [39]. In addition, several studies quantify total circulating miRNAs rather than EV-associated miRNAs. Therefore, their findings should be described as circulating miRNA signatures unless EV isolation, characterization, and vesicular carrier specificity are explicitly reported.

Among the individual candidates, miR-148a-3p has shown high discriminative performance in one clinical study. In patients with T2D and DN, its expression is markedly reduced, with a receiver operating characteristic (ROC) analysis yielding an area under the curve (AUC) of 0.916 to distinguish DN from T2D without neuropathy, in addition to independent association in multivariate analysis [40]. This finding warrants external replication, ideally with defined vesicular isolation, prespecified thresholds, standardized neuropathy phenotyping, and longitudinal clinical outcomes. Until such validations are available, this marker should be interpreted as a promising serum biomarker candidate rather than an established EV-associated diagnostic test.

Other candidates have shown promising, although less consolidated, results. miR-216a and miR-377 evaluated along with their targets GAP-43 and ANGPTL4 showed differential expression in DN and ROC curves with diagnostic value [41]. In a cohort of 49 patients with T2D, miR-128a was more highly expressed in DN compared to non-DN, with possible underexpression of miR-155 and miR-499a [42]. In contrast, another whole-blood study found no significant differences in miR-128a, miR-146a, or miR-155 between groups, but did detect differences in miR-375 between DN and healthy controls [43]. This discrepancy illustrates that circulating microRNA results can vary substantially depending on the biological matrix (serum, plasma, or whole blood) and the composition of the comparator groups.

The available evidence increasingly favors the use of multi-miRNA panels over single markers. One study reported a decrease in miR-155 and miR-146a in the peripheral blood of patients with T2D, and an additional decrease, particularly, in those with DN. This may support the idea of combining miRNAs for improving discrimination compared with each marker separately [44]. Consistently, a systematic review with bioinformatics analysis identified miR-30, miR-146, and miR-199 as the miRNAs involved in pathways relevant to DN, highlighting the extracellular matrix–receptor interaction for miR-30 [39]. Therefore, the most plausible clinical value lies not in an isolated miRNA, but in integrated signatures that capture neuroinflammation, oxidative stress, glial damage, axonal loss, and pain.

The clinical biomarker literature in DN should be interpreted cautiously because most studies are small, cross-sectional, and heterogeneous in sample type, neuropathy definition, analytical platform, and normalization strategy [39,40,41,42,43,44]. ROC-derived diagnostic estimates may be inflated when obtained from discovery cohorts without independent validation. Future studies should specify the miRNA carrier, distinguish EV-associated from total circulating miRNAs, and test whether candidate panels add value beyond clinical examination, nerve conduction studies, metabolic variables, and longitudinal outcomes.

### 4.3. Therapeutic Implications and Translational Limitations

From a therapeutic perspective, the available evidence is mainly preclinical and comes from models using EVs or exosomes derived from Schwann cells, mesenchymal stem cells, and engineered preparations enriched with selected miRNAs [32,33,34,45,46,47,48,49]. In animal models of diabetes, these strategies have improved nerve conduction velocity, thermal and mechanical sensitivity, intraepidermal nerve fiber density, and remyelination [34,45,46,47]. However, these findings remain preclinical and should not be interpreted as evidence that EV-based therapies are ready for clinical use in humans with DN.

Schwann cell-derived exosomes have shown the ability to attenuate peripheral neuropathy in type 2 diabetes models and to reverse alterations resulting from Dicer loss in Schwann cells [33,34]; while, mesenchymal-stem-cell-derived exosomes have demonstrated neuroprotective and proregenerative effects in murine models of diabetic neuropathy [46]. Furthermore, mesenchymal exosome preparations enriched with miR-146a have shown amplified therapeutic efficacy, reinforcing the possibility of designing exosomes with specific microRNA loading to modulate neuroinflammation and neural repair [45].

Additional EV-based models expand this therapeutic framework. Adipose-derived stem cell EVs carrying miR-130a-3p were reported to protect against diabetic peripheral neuropathy by delivering miR-130a-3p to Schwann cells, resulting in reduced apoptosis, proliferation even under high-glucose conditions, and modulation over the DNMT1/NRF2/HIF1a/ACTA1 axis [48]. In another preclinical model, plasma exosomes from healthy rats improved diabetic peripheral neuropathy features through enrichment of miR-20b-3p, modulation of Stat3 signaling, and regulation of Schwann-cell autophagy [49]. These studies support the hypothesis that EV cargo can be engineered or selected to influence glial survival, axonal support, autophagy, angiogenesis, and nerve repair in experimental models [48,49].

However, evidence from therapeutic studies demonstrates that EVs are not intrinsically beneficial. High-glucose-stimulated Schwann cell-derived exosomes can carry pathogenic miRNA cargo and promote axonal injury and neuropathy-like changes [32]. Therefore, EV-based therapy requires precise definition of the donor cell, donor-cell metabolic state, culture conditions, cargo profile, vesicle purity, and recipient-cell response. The same broad EV category may contain protective or harmful vesicles depending on the biological context.

Despite these findings, clinical translation still faces substantial challenges. It will be necessary to define the cell source, production method, vesicular purity, dosage, route of administration, biodistribution, duration of effect, immunogenicity, and long-term safety. Furthermore, given that many miRNAs have pleiotropic effects, therapeutic manipulation must demonstrate tissue specificity and the absence of off-target effects. For DN, additional challenges include achieving delivery to peripheral nerves, dorsal root ganglia, Schwann cells, vasa nervorum, or skin small fibers; as well as the avoidance of nonspecific uptake by liver, spleen, or immune cells, and demonstrating functional improvement beyond molecular target engagement. In this regard, DN represents an experimental setting for EV-based therapeutic development, but clinical translation requires standardized EV production, biodistribution studies, target engagement, safety assessment, and carefully designed early human studies [47]. 

## 5. EV-Associated and Circulating miRNAs as Early Mediators in Diabetic Kidney Disease

Diabetic kidney disease (DKD) affects a considerable proportion of people with diabetes and is a major cause of chronic kidney disease. The central clinical problem is that conventional markers, such as albuminuria and estimated glomerular filtration rate, usually identify damage when structural or functional renal impairment is already present. Therefore, miRNAs in urinary or plasma vesicles have been investigated as candidate biomarkers and potential mediators of glomerular and tubular pathogenesis [50,51].

DKD should not be understood solely as a local consequence of sustained hyperglycemia, but as a microvascular and tubulointerstitial complication in which inflammation, oxidative stress, endothelial damage, podocyte loss, and fibrosis converge. In this context, EVs may participate in communication between podocytes, glomerular endothelium, mesangial cells, and proximal tubule, potentially facilitating post-transcriptional communication between renal compartments; however, not all renal miRNA studies are EV-specific [50,51,52]. For this revision, studies were interpreted according to whether they measured EV-associated miRNAs, urinary total miRNAs, plasma total miRNAs, tissue miRNAs, or mixed extracellular RNA fractions. This distinction is essential because urinary and plasma miRNAs can be vesicle-encapsulated, Argonaute-bound, lipoprotein-associated, or derived from non-vesicular ribonucleoprotein complexes.

The available evidence allows us to organize the microRNAs involved in DKD into three functional axes [53,54,55,56,57,58,59,60,61,62,63]:

(1) Profibrotic microRNAs, such as miR-21, which promote TGF-β/Smad signaling and extracellular matrix accumulation.
(2) Antifibrotic or protective microRNAs, such as the miR-29 and miR-30 families.
(3) MicroRNAs related to inflammation and endothelial dysfunction, such as miR-155, miR-146a, miR-126, miR-221, and members of the miR-200 family.

A fourth interpretative layer should also be considered, as miRNAs and EV-miRNA signatures may reflect distinct renal or systemic compartments. Urinary EVs may provide information closer to podocyte, tubular, and urinary tract injury, whereas plasma EVs and circulating miRNAs may integrate renal, endothelial, platelet, leukocyte, hepatic and systemic inflammatory signals. Therefore, renal specificity depends not only on the miRNA analyzed but also on the biological matrix, EV isolation strategy, and clinical context. 

### 5.1. Clinical Aspects and Limitations of Conventional Markers

Albuminuria and estimated glomerular filtration rate (eGFR) remain the central clinical tools for diagnosing and monitoring DKD. However, both have significant limitations: albuminuria can fluctuate, does not always precede the decline in renal function, and may appear when glomerular or tubular damage is already established; meanwhile, a decrease in eGFR usually reflects later functional loss. Therefore, there is interest in biomarkers that capture early cellular stress, podocyte damage, tubular activation, or endothelial remodeling before irreversible injury [50,52].

The biological plausibility of this approach Is supported by the functional anatomy of the nephron. The podocyte, the fenestrated endothelium, and the glomerular basal membrane comprise a highly specialized filtration barrier; in turn, the proximal tubule receives signals derived from the ultrafiltrate and from eVs released by injured podocytes. Exposure to hyperglycemia can increase the release of podocyte eVs, and these vesicles can induce tubular apoptosis, linking early glomerular damage with inflammation and tubulointerstitial fibrosis [52,64,65]. This EV-mediated podocyte–tubule axis provides a biologically plausible mechanism by which glomerular stress may influence tubular injury, although its prognostic relevance in humans requires further validation.

This model of EV-mediated glomerulotubular communication is particularly relevant because it explains how initially focal damage can spread to other renal compartments. Thus, vesicular miRNAs could reflect not only established damage but also the biological direction of the process: inflammatory, fibrogenic, endothelial, or podocyte-mediated. Nevertheless, evidence of EV-mediated causality requires more than differential miRNA abundance in urine or plasma. Stronger evidence requires vesicle isolation and characterization, identification of the donor compartment, uptake by a recipient renal cell, transfer of miRNA cargo, repression of a validated target, and reproduction or rescue of the renal phenotype.

From a clinical perspective, urinary EV-miRNAs should be interpreted as organ–proximal candidates rather than definitive diagnostic tools. They may improve early detection, but their incremental value should be tested against albuminuria, eGFR slope, blood pressure, diabetes duration, glycemic control, renoprotective medication use, and histological or imaging correlates when available [50,52,66,67,68,69,70,71].

### 5.2. MicroRNA-Regulated Signaling Networks in DKD

The miRNAs involved in DKD do not act on an isolated pathway. Collectively, they modulate TGF-β/Smad, NF-κβ, PI3K/Akt, MAPK, and Wnt/β-catenin pathways that converge in fibrosis, inflammation, oxidative stress, cellular hypertrophy, and epithelial–mesenchymal transition [53,72,73,74].

miR-21 is interpreted as one of the most consistent profibrotic nodes. It can be induced by TGF-β1 and, in turn, promotes Smad signaling by inhibiting negative regulators such as Smad7 and PTEN, thus promoting extracellular matrix deposition and renal fibrosis [53,54]. For this reason, miR-21 represents both a candidate biomarker of fibrogenic activity and a potential therapeutic target for antifibrotic strategies. However, miR-21 should not be interpreted as uniformly pathogenic in every context without considering cell type, disease stage, carrier, and tissue compartment, because the biological effect of a miRNA may differ between podocytes, mesangial cells, tubules, immune cells, and circulating EV fractions.

Conversely, the miR-29 family functions as an antifibrotic axis by limiting collagen and fibronectin expression; its suppression by TGF-β promotes extracellular matrix accumulation and progression to tubulointerstitial fibrosis [55]. This contrast between miR-21 and miR-29 illustrates that renal progression may depend on the balance between profibrotic and counterregulatory vesicular signals. Therefore, future biomarker panels should not rely only on the absolute expression of isolated miRNAs but should consider ratios or combined profiles reflecting the balance between profibrotic and antifibrotic pathways.

The inflammatory dimension is represented by miR-146a and miR-155. miR-146a typically acts as a negative regulator of the IRAK1/TRAF6/NF-κB pathway, while miR-155 is associated with persistent inflammatory activation and JAK/STAT signaling through interactions with SOCS1 [56,57]. Taken together, these microRNAs connect DKD with mechanisms shared by other diabetic complications, especially neuropathy and retinopathy, where inflammation and endothelial dysfunction are also central axes. This shared biology is consistent with the multi-organ framework proposed in this review, but it does not establish that a single EV-miRNA axis causally links kidney, nerve, retina, and liver injury in humans.

In addition to miRNA-regulated pathways, there also exists emerging epitranscriptomic mechanisms that may contribute to DKD progression. A recent experimental study reported that methyltransferase-like 16 (METTL16), a key m6A methyltransferase, may contribute to diabetic nephropathy progression through epigenetic suppression of V-set pre-B cell surrogate light chain 3 (Vpreb3) and oxidative-stress-related mechanisms [75]. Although this evidence does not directly demonstrate EV-miRNA communication, it reinforces the concept that diabetic kidney injury is shaped by interacting post-transcriptional and epitranscriptomic networks, including miRNAs, RNA-binding proteins, and RNA modifications.

### 5.3. Specificity by Cell Compartment: Podocyte, Endothelium, and Tubule

The interpretation of microRNAs in DKD depends of the predominant cellular compartment. In podocytes, the miR-30 family is abundantly expressed under homeostatic conditions and contributes to preserving the cytoskeleton, the filtration diaphragm, and epithelial identity. Chronic exposure to hyperglycemia or TGF-β reduces the expression of this family, promoting dedifferentiation, proteinuria, and fibrosis [76,77,78].

Other miRNAs, such as miR-93, miR-217, miR-25, and miR-26a, have been associated with chromatin remodeling, oxidative stress, cellular aging, albuminuria, and CTGF regulation [79,80,81,82]. This suggests that podocyte damage does not depend on a single linear pathway, but rather on a post-transcriptional network that integrates metabolic stress, profibrotic signaling, and loss of cellular identity. When podocyte-derived EVs are evaluated, the critical question is whether their cargo reflects podocyte stress, actively transfers injury signals to tubular or endothelial cells, or both.

**Figure 2:**
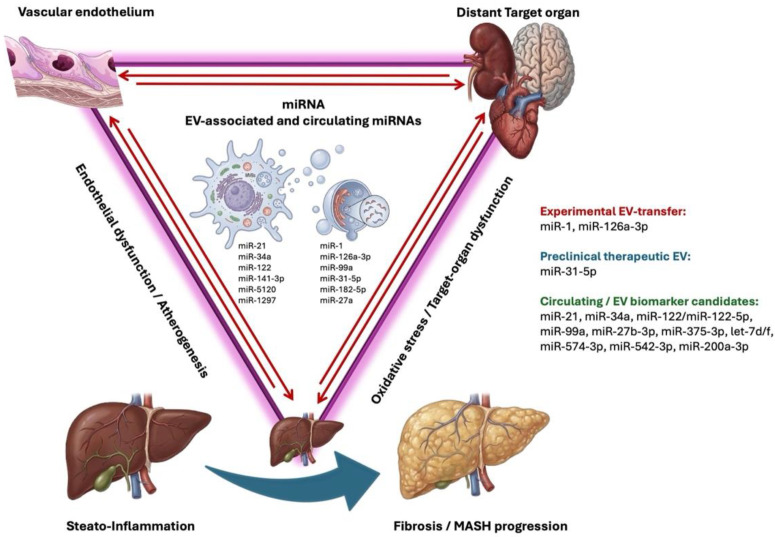
Conceptual model of the liver—endothelium—target organ axis in T2D with MASLD. Lipotoxicity and hepatic insulin resistance may modify the release and cargo of hepatocyte-derived EVs. In experimental models, hepatocyte EVs can transfer miR-1 to endothelial cells and promote inflammatory and atherogenic responses, while EVs derived from steatotic hepatocytes have been linked to β-cell dysfunction via miR-126a-3p and IRS-2 [128,129]. Circulating miR-21, miR-34a, and miR-122 have been associated with inflammation, redox stress, and fibrogenic progression in MASLD/T2D cohorts [123,130]. Recent human EV-miRNA studies suggest that EV-associated and non-vesicular miRNA compartments may provide non-equivalent biomarker information across MASLD stages [131,132,133]. This model should be interpreted as hypothesis-generating rather than as definitive proof of a unified human EV-miRNA causal axis.

In the glomerular endothelium, miR-126 maintains vascular homeostasis and physiological angiogenesis, while its decrease is associated with microvesicular dysfunction [59,60,61]. Complementarily, miR-221 and members of the miR-200 family can modulate endothelial permeability and responses related to VEGF or claudin-5 [62,83,84]. This endothelial dimension allows us to link DKD with the systemic axis of microvascular damage shared with the retina, peripheral nerves, and metabolic dysfunction-associated steatotic liver disease (MASLD), as summarized in Figure 2. However, endothelial miRNAs detected in plasma may not be kidney-specific, because they can also arise from systemic vascular injury, platelet activation, inflammation, or cardiometabolic stress.

In the tubule, miR-21, miR-155, and miR-214 tend to promote inflammation and fibrosis, while miR-29 and miR-23b act as antifibrotic counterweights by preserving epithelial characteristics and limiting extracellular matrix deposition [56,57,58,73,85,86,87,88,89,90,91]. The presence of microRNAs with opposing effects in different renal compartments reinforces the need to interpret vesicular signatures according to their probable cellular origin and not just according to their total concentration in urine or plasma.

For this reason, compartment-specific interpretation should be incorporated into future DKD studies. Urinary EVs enriched in podocyte-, tubular-, or glomerular- endothelial markers may provide greater mechanistic specificity than unfractionated urine or plasma. Conversely, plasma EV-miRNAs may be useful for identifying systemic microvascular or inflammatory phenotypes, but they should not be assumed to represent renal pathology unless validated against renal outcomes or kidney-proximal samples.

### 5.4. Urine, Plasma, and EVs as Biomarker Platforms

Urinary miRNAs have a conceptual advantage over other biomarkers, as they can directly reflect vesicular release from podocytes, tubules, and urinary tract cells. Elevations in miR-21, miR-192, and miR-29 have been described in association with proteinuria, decreased eGFR, and tubulointerstitial fibrosis. Furthermore, miR-21-5p and miR-30b-5p in urinary exosomes have been proposed as candidates for identifying early DKD, while profiles such as miR-130a, miR-145, miR-155, and miR-424 have been explored in type 1 diabetes with incipient microalbuminuria [66,67,68,69].

Recent urinary EV studies support the use of this matrix for DKD biomarker discovery, but not yet for routine clinical implementation. A multi-cohort study identified selected urinary extracellular vesicle miRNAs as candidate biomarkers for the early diagnosis of DKD. The findings were replicated across independent cohorts and publicly available datasets, while preanalytical variables, including urine collection method and centrifugation before storage, were also evaluated. [52]. In addition, urinary exosomal miR-136-5p was reported to be elevated in patients with DKD compared with patients with diabetes without DKD, with a diagnostic performance in ROC analysis and correlation with UACR, cystatin C, eGFR, and CKD progression-risk indicators [92]. These findings support urinary EV-miRNAs as organ-proximal candidates, but they remain insufficient for routine clinical implementation without larger longitudinal and externally validated cohorts.

Plasma evidence is harder to interpret as it reflects systemic sources and have greater preanalytical variability. Even so, studies in plasma EVs have identified miR-99a-5p as a possible marker linked to macroalbuminuria and podocyte protection via mTOR [70]. There is a marked difference between urine and plasma biomarkers. For instance, urine biomarkers may offer greater renal specificity, while plasma can capture systemic signals from inflammation, endothelium, and metabolism, although with less tissue specificity.

Finally, the distinction between EV-associated miRNAs and total circulating miRNAs is especially important in DKD. Some longitudinal human evidence supports the prognostic value of circulating miRNAs, but not necessarily their EV localization. For example, circulating TGF-β1-regulated miRNAs, including let-7c-5p, miR-29a-3p, let-7b-5p, and miR-21-5p, were associated with rapid progression to ESRD in a prospective cohort of proteinuric patients with type 1 diabetes that were followed for 7–20 years [93]. This study is valuable because it addresses longitudinal renal progression, but its results should be interpreted as circulating-miRNA evidence rather than EV-specific evidence unless vesicular carrier status is demonstrated.

Translating these findings into clinical practice will require standardized protocols for sample collection and storage, EV isolation, RNA extraction, normalizer selection, and miRNA quantification. In addition, multicenter longitudinal studies with clinically relevant renal outcomes are needed to determine whether vesicular miRNA panels provide incremental predictive or diagnostic value beyond established variables, including albuminuria, eGFR and CKD stage progression, blood pressure, diabetes duration and glycemic control, cardiovascular events, and response to different therapies. [66,70,71]. 

Registered human studies evaluating urinary exosomes in T2D-related DKD indicate that the field is moving toward structured biomarker validation, although these studies should not be interpreted as evidence of clinical utility until results are reported and externally validated [92]. However, these studies should be interpreted as biomarker-development efforts rather than proof of clinical implementation or therapeutic efficacy. Future studies should be longitudinal, multicenter, and powered to determine whether urinary EV-miRNA panels predict renal outcomes beyond conventional clinical variables and whether they retain performance across sex, diabetes type, ethnicity, medication exposure, albuminuria category, and eGFR stage.

Overall, DKD is a clinically accessible setting for urinary EV-miRNA biomarker research because urine provides a kidney-proximal matrix and current markers do not fully capture early cellular injury [50,51,52,66,67,68,69,70,71].

## 6. EV-Associated and Circulating miRNAs in Diabetic Retinopathy: From Early Neurovascular Damage to Angiogenesis

Diabetic retinopathy (DR) is a progressive complication that combines microvascular damage, retinal neurodegeneration, inflammation, endothelial dysfunction, and disruption of the blood–retinal barrier. Although fluorescein angiography, fundus photography, and optical coherence tomography are essential clinical tools; they are mainly used to identify established structural changes. Therefore, circulating, vitreous, and EV-associated miRNAs have generated interest as molecular signals that may complement retinal imaging and reflect neurovascular stress in selected settings [94,95].

The pathophysiology of DR integrates chronic hyperglycemia, activation of the polyol pathway, oxidative stress, PKC activation, accumulation of advanced glycation end products (AGEs), inflammation, pericyte apoptosis, endothelial injury, disruption of the blood–retinal barrier, ischemia, and activation of the VEGF/HIF-1 axis [96,97,98,99]. In parallel, the neural retina may exhibit ganglion cell apoptosis, Müller cell dysfunction, and microglial activation before the appearance of advanced vascular lesions. This sequence justifies interpreting DR as a neurovascular disease and not merely as a terminal microangiopathy. Accordingly, EV-miRNA studies in DR should be interpreted within the retinal neurovascular unit, including retinal endothelial cells, pericytes, Müller glia, microglia, retinal pigment epithelial cells, ganglion cells, and infiltrating immune cells.

In this scenario, miRNAs can act as regulators of oxidative, inflammatory, angiogenic, and barrier integrity pathways. For example, miR-146a can limit IRAK1/TRAF6/NF-κβ signaling and function as an anti-inflammatory brake, while miR-155 and miR-21 have been linked to inflammatory amplification, barrier damage, and fibrosis. miR-124 has been associated with the regulation of microglial activation, and miR-126 and miR-200b are involved in endothelial homeostasis, VEGF signaling, and vascular permeability [100,101,102,103,104,105,106]. However, associations between miRNA expression and DR stage should not be interpreted as causal evidence unless vesicular transfer, recipient-cell uptake, target repression, and functional rescue have been demonstrated.

### 6.1. Early Neurovascular Damage and the Need for Preclinical Biomarkers

DR should be considered a neurovascular disease from its earliest stages. Before evident changes are present in fundus examination, angiography, or optical coherence tomography, pericyte apoptosis, endothelial dysfunction, Müller cell activation, microglial polarization, and ganglion cell loss can occur [96,97,98,99]. This preclinical phase is particularly relevant because it represents a potential window for biomarkers and preventive strategies before progression to macular edema, ischemia, or neovascularization.

Chronic hyperglycemia activates multiple metabolic pathways that converge on oxidative stress, inflammation, and vascular dysfunction. The polyol pathway, AGEs, PKC activation, and increased VEGF contribute to blood–retinal barrier disruption, macular edema, ischemia, and neovascularization [107,108,109,110,111]. The added value of miRNAs lies in their ability to modulate several nodes in these networks, helping to explain why retinal damage can progress differently among patients with seemingly similar glycemic exposures.

From a vesicular perspective, EVs released by pericytes, retinal endothelial cells, Müller cells, microglia, or neural retina could participate in local intercellular communication related to inflammation, barrier dysfunction, and angiogenesis; although EV-mediated transfer has not been demonstrated for all candidate miRNAs [95,100,101,102,103]. However, many clinical studies in DR quantify total circulating miRNAs and do not distinguish whether these are encapsulated in EVs, bound to Argonaute proteins, or associated with lipoproteins. This distinction is crucial for interpreting whether the microRNA functions primarily as a circulating biomarker, an intercellular mediator, or both.

Donor cell-specific EV communication is particularly important in DR. Müller glia-derived exosomal miR-9-3p has been shown to promote angiogenic behavior by targeting S1P1 and modulating VEGFR2 signaling in retinal endothelial cells, supporting a preclinical mechanism by which glial EV cargo may influence endothelial angiogenic behavior [112]. Conversely, retinal pigment epithelial cell derived exosomes enriched in miR-202-5p can suppress high glucose-induced endothelial-to-mesenchymal transition through TGFβR2/TGFβ/Smad signaling, suggesting that some retinal EV cargos may exert protective or compensatory effects in experimental models [113]. These findings illustrate that retinal EVs are not uniformly pathogenic or protective; their effects depend on donor cell type, metabolic state, vesicular cargo, recipient cell, and disease stage.

Matrix selection strongly influences interpretation. Vitreous humor is closer to the retinal microenvironment and may better reflect signals from retinal endothelial cells, pericytes, Müller cells, microglia, retinal pigment epithelium, and neural retina. However, vitreous sampling is invasive and usually restricted to patients undergoing vitrectomy, which enriches studies for advanced disease. Serum and plasma are clinically accessible but have lower retinal specificity and may reflect systemic inflammation, endothelial dysfunction, renal disease, hepatic steatosis, platelet activation, or glycemic exposure. Therefore, circulating miRNA or EV-miRNA panels should ideally be interpreted together with retinal imaging, disease stage, macular edema status, anti-VEGF exposure, diabetes duration, HbA1c, renal function and EV-carrier characterization.

### 6.2. Oxidative Stress, Inflammation, and Angiogenesis as Axes Regulated by MicroRNAs

Oxidative stress is one of the central mechanisms of retinal damage in diabetes. miR-26a-5p has been interpreted as protective in Müller cells exposed to high glucose levels by increasing antioxidant systems such as superoxide dismutase and catalase, while the reduction in miR-296-5p may be associated with increased lipid peroxidation and loss of vascular integrity [95,101,102,103]. These findings suggest that the progression of DR may depend on the balance between protective antioxidant microRNAs and microRNAs that promote inflammatory or vascular damage.

In the inflammatory axis, miR-146a typically acts as a negative regulator of the IRAK1/TRAF6/NF-κβ pathway; so, its reduction could facilitate activation of innate immunity and persistent inflammation. miR-155, on the other hand, has been associated with proinflammatory responses, microglial activation, and barrier damage. miR-21 may be involved in inflammation, fibrosis, and PTEN/PPAR-α signaling, while miR-124 could modulate microglial activation toward less inflammatory phenotypes [100,101,102,103]. These miRNAs link DR to mechanisms shared by neuropathy and diabetic kidney disease, particularly inflammation, oxidative stress, and endothelial dysfunction.

EV-focused inflammatory mechanisms have also been explored. Serum-derived exosomal miRNA-3976 was reported to be increased in early DR and was evaluated in retinal ganglion-like and endothelial cell models, with effects related to NF-κβ-associated mechanisms [114]. Although this study supports the concept that circulating exosomal miRNAs may participate in early retinal neurovascular stress, its small sample size and restricted population mean that miRNA-3976 should be considered a preliminary candidate rather than a validated clinical biomarker.

The transition from non-proliferative to proliferative retinopathy is related to hypoxia, HIF-1 signaling, and VEGF activation. In this axis, miR-126 is relevant due to its role in endothelial homeostasis and physiological angiogenesis; its decrease may promote vascular instability and loss of endothelial protection [105]. On the other hand, miR-200b regulates VEGF-A and vascular permeability, although its concentrations can change depending on the stage of the disease [104,106]. This dynamic pattern should be highlighted to avoid a linear interpretation of the biomarkers: the same microRNA can have different meanings in early, compensatory, or proliferative stages of DR.

Additional EV-associated miRNAs may help refine the angiogenic and macular edema phenotype. Serum exosomal miR-377-3p has been proposed as a biomarker for diabetic macular edema and was shown to regulate VEGF expression in retinal pigment epithelial cells [115]. Serum EV-encapsulated miR-431-5p has also been proposed as a biomarker for proliferative diabetic retinopathy [116]. These findings support EV-miRNAs as preliminary retinal biomarker candidates, but they require replication in larger, longitudinal, externally validated cohorts with standardized EV isolation, retinal staging, treatment-status reporting, and adjustment for systemic diabetic complications [114,115,116].

Therapeutically, preclinical work suggests that EVs may be explored as delivery platforms for protective miRNAs in retinal models. For example, bone marrow mesenchymal stem cell-derived exosomal miR-486-3p has been reported to protect against diabetic retinopathy through repression of the TLR4/NF-κβ axis [117]. Nevertheless, these findings remain preclinical and should not be interpreted as evidence that EV-based miRNA therapy is ready for routine clinical use in DR.

### 6.3. Diagnostic Stratification and Vitreous Microenvironment

As biomarkers, miRNAs in DR are promising but remain investigational, and the evidence is heterogeneous. miR-126 has been described as decreased in plasma or vitreous humor and is often interpreted as a sign of loss of endothelial protection [105]. Other candidates such as miR-21, miR-181c, and miR-1179 have been associated with progression or proliferative disease in specific studies [118,119,120]. However, these associations depend on the matrix analyzed, the DR stage, the presence of macular edema, the type of comparator, and the quantification method.

The available evidence suggests that multi-miRNA panels may be more informative than isolated markers, although standardized panels have not yet been established. Combinations including miR-21, miR-181c, and miR-1179 have shown the ability to discriminate between non-proliferative and proliferative retinopathy in specific studies, while miR-126 has been evaluated as a microvascular risk marker in plasma and vitreous humor [104,105,118]. This panel-based approach is consistent with the pathophysiology of DR, because inflammation, hypoxia, angiogenesis, neural damage, and barrier dysfunction develop simultaneously.

However, the diagnostic literature remains limited by small cohorts, cross-sectional designs, inconsistent staging criteria, variable miRNA platforms, heterogeneous normalization strategies, and incomplete adjustment for diabetes duration, HbA1c, kidney disease, hypertension, dyslipidemia, anti-VEGF therapy, laser treatment, and macular edema. ROC curves derived from small discovery cohorts may overestimate biomarker performance, especially when external validation and prespecified thresholds are lacking. Therefore, diagnostic performance should be interpreted as preliminary unless replicated in independent cohorts and shown to add value beyond retinal imaging and conventional clinical variables.

Vitreous humor provides a closer reading of the ocular microenvironment and can capture local signals from the neural retina, endothelium, pericytes, and glial cells. However, its collection is invasive and generally limited to patients undergoing vitrectomy, thus having less utility for population screening or early detection. In contrast, serum and plasma are more accessible, but they have lower tissue specificity and may reflect systemic sources of miRNAs associated with nonspecific inflammation, metabolism, or vascular damage [105,118,119,120]. Therefore, a clinically viable strategy could combine circulating panels with ophthalmological, metabolic, and imaging variables, reserving vitreous analysis for mechanistic studies or advanced disease.

The vitreous compartment is particularly useful for mechanistic interpretation. Vitreous miRNA profiling in proliferative diabetic retinopathy has shown that ocular miRNA signatures may differ according to disease activity and anti-VEGF exposure [120], while systematic evaluation of vitreous miRNAs supports their potential as disease-proximal biomarkers in retinal disorders [119]. Nevertheless, vitreous studies are often enriched for severe disease and may not represent early non-proliferative DR. Therefore, vitreous miRNAs should be interpreted as local mechanistic or advanced-disease signals rather than as population-level screening tools.

Human EV-focused validation is beginning to emerge. Registered studies evaluating exosome changes in proliferative diabetic retinopathy and plasma exosome proteomics across DR stages indicate that the field is moving toward clinical validation of EV-based biomarkers [121,122]. These studies should be described as translational biomarker-development efforts, not as evidence of established EV-based clinical tests or EV-based therapies.

Overall, DR represents a setting in which circulating, vitreous, and EV-associated miRNAs may help characterize neurovascular stress, angiogenesis, and disease stage, but current evidence is not sufficient for clinical implementation. Future studies should separate EV-associated from total circulating miRNAs, report retinal stage and treatment exposure, and test incremental value over fundus photography, OCT/OCT-A, fluorescein angiography, and conventional clinical variables [94,105,112,113,114,115,116,117,118,119,120,121,122].

## 7. EV-Associated and Circulating miRNAs in Diabetes-Associated MASLD: Liver-Vessel-Target Organ Axis

The coexistence of MASLD and T2D is frequent and clinically relevant [123,124,125]. In this context, the liver should not be considered solely a target organ of insulin resistance, but also a potential source of systemic signals [124,125]. Under lipotoxicity, inflammation, and endoplasmic reticulum stress, the hepatocyte modifies its transcriptome, its post-transcriptional signaling, and the cargo released in extracellular vesicles, supporting the concept of a liver—endothelium—target organ axis [125,126,127].

Unlike other more classically microvascular diabetic complications, MASLD may contribute an additional systemic dimension: the steato-inflammatory liver can release pro-inflammatory, pro-fibrotic, and metabolic signals that may influence cardiorenal risk and distant organ function. This conceptual model is summarized in Figure 2, which illustrates how hepatic lipotoxicity and insulin resistance favor the release of EVs enriched in microRNAs with potential impact on endothelium, pancreatic β cell and target tissues. However, the proposed liver–endothelium–target organ axis should be interpreted as a biologically plausible and hypothesis-generating framework. Direct empirical evidence linking MASLD, neuropathy, diabetic kidney disease, and retinopathy through a single EV-miRNA axis in the same human cohorts remains limited.

### 7.1. MASLD as a Systemic Node of Metabolic Communication

The usefulness of interpreting MASLD as a systemic node lies in its ability to integrate three levels of evidence: hepatocyte signaling via lipotoxicity, vesicular communication with target organs, and circulating miRNA signatures with biomarker potential. In experimental models, EVs derived from palmitate-exposed hepatocytes can transfer miR-1 to endothelial cells, activate vascular inflammation, and promote atherogenesis; furthermore, miR-1 inhibition by antagomiR attenuates this phenotype [128]. This causal chain is particularly relevant because it connects metabolic stimulus, vesicular vehicle, miRNA loading, receptor tissue, and vascular outcome.

This liver—endothelium model represents a well-described preclinical example in MASLD-related EV research because it links donor-cell stress, EV cargo, recipient-cell response, and vascular phenotype [128]. Nevertheless, it remains primarily experimental. Extrapolation to chronic human T2D requires caution because circulating EVs in patients may originate not only from hepatocytes but also from adipocytes, platelets, leukocytes, endothelial cells, skeletal muscle, kidney, and other tissues affected by obesity, insulin resistance, inflammation, and vascular disease. Therefore, hepatocyte-derived EV-miRNAs should be interpreted as candidate mediators of vascular injury in experimental models rather than as validated human causal biomarkers.

The liver-pancreas axis is also relevant. EVs derived from steatotic hepatocytes have been linked to β-cell apoptosis and worsening diabetes via miR-126a-3p, with targets such as IRS-2 [129]. This finding supports the idea that MASLD may be involved in the natural history of T2D not only as a hepatic comorbidity but also as a modulator of pancreatic β-cell dysfunction. However, extrapolation to humans requires caution because beta cell physiology in T2D also depends on chronic glyco-lipotoxicity, genetic background, systemic inflammation, disease duration, treatment, and residual pancreatic reserve [129,134].

Donor cell-specific EV communication is therefore central to the interpretation of MASLD in diabetes. Hepatocyte-derived EVs may influence endothelial cells, pancreatic β-cells, macrophages, hepatic stellate cells, and potentially distant vascular beds. Macrophage-derived exosomes may modulate cardiometabolic inflammation and diabetes in obesity [134], while MSC-derived EVs may preferentially accumulate in the liver and modify hepatic macrophage signaling in experimental T2D with fatty liver disease [135]. These observations indicate that EV effects in MASLD are strongly dependent on donor cell identity, metabolic state, EV cargo, recipient cell type, and disease stage.

Taken together, the miR-1 and miR-126a-3p models provide a mechanistic framework for liver-vasculature-pancreas communication. Although these data do not yet establish causality in humans with chronic T2D, they do warrant evaluating whether hepatic vesicular signatures can predict vascular risk, metabolic progression, or β-cell dysfunction in individuals with MASLD and diabetes. Future studies should therefore distinguish hepatocyte-derived EVs from other circulating EV populations and should test whether these signals are associated with endothelial dysfunction, β-cell decline, cardiovascular events, renal progression, retinopathy, or neuropathy beyond conventional metabolic risk factors.

### 7.2. Inflammation, Fibrosis, and Emerging EV-miRNA Biomarkers

During the transition from simple steatosis to steatohepatitis/fibrosis, miR-21, miR-34a, and miR-122 repeatedly appear as nodes of inflammation, redox stress, and fibrogenic remodeling [125,130]. These microRNAs should not be interpreted solely as isolated liver markers, but rather as part of a broader metabolic and inflammatory signature that could link MASLD with vascular, renal, and systemic risk in T2D.

Clinically, serum miR-99a has been proposed as a biomarker for MASLD in people with T2D due to its association with HbA1c, IL-6, and mTOR [123]; likewise, miR-141-3p and ZMPSTE24/prelamin suggest non-canonical pathways related to nuclear architecture and steatosis severity [136]. These findings broaden the interpretation of MASLD beyond the classic pathways of lipotoxicity and inflammation, incorporating mechanisms of nuclear architecture, cellular stress, and post-transcriptional remodeling.

Human EV-miRNA evidence in MASLD is emerging and should be incorporated cautiously as biomarker-level evidence. In a cohort of biopsy-proven MASLD, circulating EV miRNome profiling identified miR-27b-3p, miR-30a-5p, miR-122-5p, miR-375-3p, miR-103a-3p, let-7d-5p, and let-7f-5p as differentially expressed according to steatohepatitis and significant fibrosis, linking EV-miRNA signatures to lipid metabolism, adipose tissue insulin resistance, inflammatory pathways, and “at-risk MASH” [131]. This suggests that EV-miRNAs may capture aspects of metabolic and inflammatory disease activity, but external validation is required before clinical use.

Additional EV-focused biomarker studies suggest that serum EV panels may improve non-invasive detection of MASLD. A panel including EV-associated miR-574-3p, miR-542-3p, and miR-200a-3p was reported to be elevated in serum EVs from patients with MASLD and proposed as a candidate non-invasive biomarker set [132]. Similarly, evaluation of EV characteristics and miRNA transport across the MASLD spectrum suggested that miRNA abundance may differ between EVs and total serum, reinforcing the need to distinguish vesicular from non-vesicular miRNA compartments [133]. These findings directly address the limitation that many earlier MASLD studies measured total circulating miRNAs without defining the molecular carrier.

A critical point is distinguishing whether these signatures represent markers of liver status, mediators of systemic damage, or both [125]. This distinction is especially important because many studies quantify total circulating microRNAs and do not always identify whether the microRNA is encapsulated in EVs, bound to Argonaute proteins, or transported by lipoproteins. Therefore, biomarker interpretation must consider the molecular carrier, the likely tissue of origin, the stage of MASLD, the presence of T2D, and the clinical outcomes assessed.

Matrix selection is particularly important in MASLD. Serum and plasma are clinically accessible but may reflect liver injury, adipose tissue inflammation, platelet activation, endothelial dysfunction, kidney disease, cardiovascular disease, or systemic metabolic stress. Liver tissue provides mechanistic specificity but is invasive and rarely available for longitudinal monitoring. EV-enriched fractions may improve biological interpretability, but only if vesicle isolation, characterization, purity, RNA extraction, and normalization are adequately reported. Therefore, MASLD EV-miRNA studies should be interpreted according to sample type, EV isolation method, analytical platform, disease stage, presence of T2D, fibrosis assessment, and whether longitudinal outcomes were measured.

At present, no standardized MASLD EV-miRNA panel has been validated for routine clinical use. The most promising strategy is not to rely on a single miRNA, but to develop multi-miRNA panels integrated with liver enzymes, metabolic markers, fibrosis scores, elastography, imaging-based steatosis quantification, glycemic control, inflammatory markers, and cardiovascular or renal risk variables. Such integrated approaches are more consistent with the systemic biology of MASLD in T2D.

### 7.3. Hepatic Interventions, Therapeutic EVs, and Multi-Organ Impact

The therapeutic dimension of MASLD associated with T2D has systemic implications, and extends beyond the liver as an isolated organ; interventions targeting hepatic metabolism may reshape EV-mediated signaling and influence tissues involved in cardiometabolic health and diabetic complications. In a T2D model with MASLD, EVs derived from umbilical cord mesenchymal stromal cells showed predominant hepatic accumulation and delivered miR-31-5p, leading to suppression of PDGFB in hepatic macrophages, and improvement in the hepatic phenotype. In parallel, the study also reported restoration of hippocampal pericytes and neurovascular function [135]. Although this study does not demonstrate that MASLD is the primary cause of microvascular damage, it does support the idea that modulating the hepatic microenvironment can affect distant vascular phenotypes.

This preclinical therapeutic model is relevant for EV-based translation because it suggests that therapeutic EVs may influence hepatic and extrahepatic phenotypes, while also illustrating key barriers to human application: donor cell selection, EV purity, cargo reproducibility, liver targeting, dose, biodistribution, route of administration, long-term safety, immune response, and off-target gene regulation. Therefore, therapeutic EVs in MASLD-associated diabetes should be presented as a promising preclinical strategy rather than as a clinically validated intervention.

From a shared miRNA perspective, miR-483-5p has been proposed as a candidate linked to T2D, MASLD, diabetic nephropathy, and neurological injury [137]. This type of shared post-transcriptional node is useful for constructing a multi-organ communication model in diabetes, where the liver, kidney, peripheral nerve, retina, and endothelium do not function as isolated compartments, but rather as tissues connected by vesicular, inflammatory, and metabolic signals.

Other therapeutic and metabolic studies support the concept that hepatic miRNA networks are modifiable. miR-423-5p has been linked to hepatic gluconeogenesis and hyperglycemia through the FAM3A–ATP–Akt pathway [138], semaglutide has been associated with improvement in hepatic steatosis in T2D/NAFLD mice via a miR-5120/ABHD6-related mechanism [139], miR-182 has been proposed as a link between T2D and fatty liver disease in obesity [140], and obesity-associated exosomal miRNAs have been shown to modulate glucose and lipid metabolism in mice [141]. These findings suggest that miRNA pathways may be responsive to metabolic interventions, but they do not establish validated EV-miRNA therapeutic targets in humans.

Nevertheless, direct human evidence linking MASLD to neuropathy, diabetic kidney disease, and retinopathy through shared EV-miRNA transfer remains limited. Current evidence is strongest for: (i) experimental hepatocyte-to-endothelium transfer of mir-1 [128]; (ii) experimental steatotic hepatocyte-to-β-cell transfer of miR-126a-3p [129]; (iii) circulating or serum EV-miRNA associations with MASLD activity and fibrosis [131,132,133]; and (iv) preclinical therapeutic EV models targeting the hepatic microenvironment [135]. Integrated human studies measuring MASLD severity, circulating EV-miRNAs, organ-proximal miRNAs, retinal imaging, kidney outcomes, neuropathy phenotyping, and longitudinal clinical progression in the same individuals are needed to determine whether these associations reflect shared systemic stress, organ-specific pathology, or true interorgan communication [125,131,132,133].

**Figure 3:**
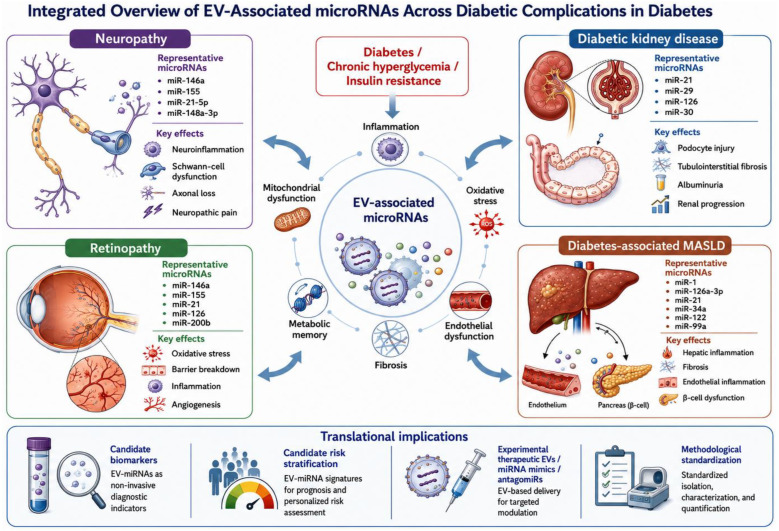
Multi-organ integration of EV-associated microRNAs in diabetic complications. Chronic hyperglycemia, insulin resistance, oxidative stress, low-grade inflammation, mitochondrial dysfunction, endothelial dysfunction, and metabolic memory may modify EV release and EV cargo in different tissues. EV-associated miRNAs have been linked to post-transcriptional networks in target organs, although functional mediation requires demonstration of vesicular uptake and target engagement. In DN, miRNAs such as miR-146a, miR-155, miR-21-5p, and miR-148a-3p have been associated with neuroinflammation, Schwann-cell dysfunction, axonal loss, and neuropathic pain [26,27,28,29,30,31,32,33,34,35,36,37,38,39,40,41,42,43,44,45,46,47,48,49]. In DKD, miR-21, miR-29, miR-126, and miR-30 have been associated with podocyte injury, tubulointerstitial fibrosis, albuminuria, and renal progression [50,51,52,53,54,55,56,57,58,59,60,61,62,63,64,65,66,67,68,69,70,71,72,73,74,75,76,77,78,79,80,81,82,83,84,85,86,87,88,89,90,91,92,93]. In DR, miR-146a, miR-155, miR-21, miR-126, and miR-200b have been linked to oxidative stress, barrier disruption, inflammation, and angiogenesis [94,95,96,97,98,99,100,101,102,103,104,105,106,107,108,109,110,111,112,113,114,115,116,117,118,119,120,121,122]. In diabetes-associated MASLD, miR-1, miR-126a-3p, miR-21, miR-34a, miR-122, and miR-99a have been associated with liver inflammation, fibrosis, endothelial inflammation, and β-cell dysfunction [123,124,125,126,127,128,129,130,131,132,133,134,135,136,137,138,139,140,141]. The figure summarizes the translational potential of EV-associated miRNAs as candidate biomarkers, risk-stratification tools, therapeutic targets, and experimental delivery platforms. The model should be interpreted as hypothesis-generating rather than as definitive proof of a unified EV-miRNA causal axis in humans.

Table 1 summarizes the miRNAs associated with major diabetes-related complications, including MASLD, DN, DKD, DR, together with their biological sources, target tissues, proposed functional effects, levels of evidence, and translational limitations. By organizing the available findings across hepatic, neural, renal, and retinal axes, this synthesis enables the identification of both shared an tissue-specific EV-miRNA signaling patterns, while also highlighting relevant gaps in mechanistic and clinical validation. Supplementary Table S1 further documents the methodological heterogeneity among studies evaluating EV-associated and total circulating miRNAs. Building on this comparative framework, Figure 3 presents an integrative, hypothesis-generating model in which EV-mediated miRNA signaling may contribute to interorgan communication and to the development and progression of multisystem diabetic complications, without implying that the proposed pathways have been causally established.

## 8. Clinical and Translational Perspectives of Extracellular Vesicle-Associated microRNAs in Diabetes

The reviewed evidence suggests that EV-associated miRNAs represent an investigational platform with potential biomarker and mechanistic relevance. They may function as biomarkers of tissue stress, disease activity, or risk stratification, and in experimental settings, they may act as functional mediators when vesicular transfer, recipient-cell uptake, target repression, and phenotypic effects are demonstrated [13,17,23,24]. This dual nature is particularly relevant in diabetes, where chronic complications are not solely explained by sustained hyperglycemia, but also by dynamic communication networks between adipose tissue, liver, endothelium, kidney, retina, peripheral nerves, and pancreatic islets. The main miRNAs, biological matrices or vesicular sources, proposed mechanisms, evidence levels, biomarker or therapeutic potential, and translational limitations are summarized in Table 1, while methodological heterogeneity is detailed in Supplementary Table S1.

### 8.1. Biomarker Versus Functional Mediator: Interpreting the Level of Evidence

A central interpretative issue is the distinction between EV-associated miRNAs as biomarkers and EV-associated miRNAs as functional mediators. A miRNA detected in serum, plasma, urine, vitreous humor, or tissue may reflect disease activity without necessarily contributing to pathogenesis. Evidence of mediation requires additional experimental support, including vesicle release from a defined donor cell, uptake by a relevant recipient cell, transfer of miRNA cargo, repression of a validated target, reproduction of the phenotype by miRNA gain-of-function, attenuation by miRNA inhibition, and ideally rescue experiments. Therefore, throughout this review, clinical associations are interpreted as biomarker-level evidence unless functional vesicular transfer has been demonstrated [11,14,19,20,21].

From a diagnostic perspective, EV-associated miRNAs may offer conceptual advantages over total circulating miRNAs, but these advantages require analytical and clinical validation. Their encapsulation in EVs promotes stability in biofluids and can provide information about the tissue of origin, the functional state of the donor cell, and the molecular context in which the signal is released [11,14,20,21]. This characteristic is particularly attractive in DKD, where urinary miRNAs associated with EVs may provide kidney-proximal information related to podocyte, tubular, or glomerulotubular injury [50,51,52,66,67,68,69,70]. In DR, serum, plasma, or vitreous miRNAs could complement imaging techniques by providing molecular information related to retinal neurovascular stress [94,95,105,118,119,120]. In DN, circulating panels could contribute to detecting inflammatory, painful, or regenerative subphenotypes [37,38,39,40,41,42,43,44]. In diabetes-associated MASLD, miRNA signatures may reflect steatosis, inflammation, fibrosis, or systemic cardiometabolic risk in selected cohorts [123,125,130,136].

However, the clinical translation of these biomarkers requires abandoning the search for a single universal miRNA. The available evidence favors an approach based on multi-miRNA panels, integrated with clinical, biochemical, imaging, and, potentially, omics variables [39,40,44,66,67,68,69,70,105,118,119,120]. This approach is consistent with the pathophysiology of diabetic complications: a single miRNA can be involved in different organs, and a single complication can depend on multiple molecular pathways. Therefore, rather than interpreting each miRNA as an isolated marker, it is necessary to evaluate expression patterns capable of discriminating risk, progression, inflammatory activity, fibrosis, neurodegeneration, or therapeutic response.

In terms of personalized medicine, EV-associated miRNAs could contribute to identifying patients with greater susceptibility to specific complications despite comparable levels of glycemic control. This possibility is relevant for explaining the clinical heterogeneity observed in diabetes, as people with similar metabolic exposures may develop different degrees of microvascular and macrovascular complications. In this context, they could function as “molecular readouts” of tissue vulnerability, metabolic memory, and persistent inter-organ communication [13,14,15,16,17,22,23,24]. In the future, an integrated EV miRNA profile could be used to classify patients according to the predominance of neuroinflammatory, endothelial, fibrogenic, angiogenic, or hepato-metabolic damage, as schematically proposed in Figure 3.

### 8.2. Standardization Requirements for EV-miRNA Studies

Before clinical implementation, EV-miRNA studies in diabetic complications should adopt minimum reporting standards for sample collection, EV isolation or enrichment, vesicle characterization, RNA extraction, normalization, analytical platform, and statistical workflow [11,19,20,21]. Without these elements, differences in miRNA expression may reflect preanalytical or analytical variability rather than true biological differences. Detailed methodological domains are summarized in Supplementary Table S1.

The therapeutic potential of this platform is equally relevant, although it is still predominantly in a preclinical stage. In DN, models using exosomes derived from Schwann cells, mesenchymal stem cells, or preparations enriched with protective miRNAs have shown effects on remyelination, nerve conduction, mechanical or thermal sensitivity, and nerve fiber density [33,34,45,46,47]. Likewise, strategies using miRNA mimetics or inhibitors, such as neuroprotective miRNA replacement or pro-nociceptive miRNA blockade, suggest that targeted modulation of these molecules can modify functional phenotypes without necessarily altering baseline hyperglycemia [28,33,34,35,36,37,39,40,44,45,46].

In DR, miRNA-based therapeutic strategies face the challenge of delivering unstable, negatively charged molecules with pleiotropic potential to an anatomically protected tissue. Therefore, delivery platforms (including therapeutic EVs, lipid nanoparticles, or polymeric systems) have been proposed as potentially necessary to improve stability, bioavailability, and tissue penetration. The possibility of modulating alternative pathways to the VEGF axis, such as inflammation, ferroptosis, fibrosis, or blood–retinal barrier integrity, could be particularly useful in patients with an incomplete response to conventional anti-VEGF therapies [95,100,101,102,103,104,105,106].

In DKD, therapeutic strategies could be aimed at restoring protective miRNAs or inhibiting profibrotic miRNAs. Inhibition of axes such as miR-21/TGF-β/Smad or restoration of antifibrotic families such as miR-29 represent conceptual examples of interventions targeting renal fibrosis [53,54,55]. However, the kidney presents unique challenges: glomerular and tubular compartmentalization, urinary filtration, uptake by non-target cells, and the need to avoid unwanted systemic effects. Therefore, any therapeutic strategy based on miRNAs must demonstrate cell selectivity, long-term safety, and functional benefit beyond intermediate molecular changes [66,70,71].

In diabetes-associated MASLD, the therapeutic dimension takes on a systemic character. The liver can act as a signaling node for EV-associated molecular cargo directed to the endothelium, pancreas, kidney, and other target organs [124,125,135,137]. In experimental models, EVs derived from lipotoxic hepatocytes can transfer miRNAs capable of inducing endothelial inflammation or β-cell dysfunction, as exemplified by miR-1 and miR-126a-3p [128,129]. Figure 3 summarizes this liver—endothelium—target organ axis, while Figure 2 integrates MASLD with neuropathy, diabetic kidney disease, and retinopathy within a common network of interorgan communication mediated by EVs. Conversely, interventions that modify the hepatic environment (including antidiabetic drugs, weight loss, or therapeutic EVs) could reprogram miRNA axes and reduce profibrotic or proinflammatory signals [135,136,137]. This line of research is particularly attractive because it connects the treatment of MASLD to the prevention of vascular and microvascular complications in diabetes.

### 8.3. Proposed Roadmap for Clinical Validation

A practical roadmap for clinical validation should include three sequential levels. First, analytical validation should establish reproducibility of EV isolation, miRNA extraction, and quantification across laboratories. Second, biological validation should confirm that candidate miRNAs are associated with plausible donor cells, recipient tissues, and disease mechanisms. Third, clinical validation should test whether EV-miRNA panels improve prediction of incident or progressive neuropathy, diabetic kidney disease, diabetic retinopathy, or MASLD beyond established clinical variables. Ideally, future studies should be multicenter, longitudinal, externally validated, and designed around clinically meaningful outcomes rather than discovery-only differential expression.

Despite these opportunities, clinical translation faces significant methodological barriers. First, EV nomenclature and characterization remain inconsistent, because several studies use the terms “exosomes”, “microvesicles”, and “EV” interchangeably without sufficiently demonstrating biogenesis, size markers or purity [2,10,11,12,19]. Second, pre-analytical variability, including sample type, anticoagulant, processing time, storage temperature, freeze–thaw cycles, hemolysis, and RNA extraction method, can substantially affect the results [20,21]. Third, analytical heterogeneity related to ultracentrifugation, commercial precipitation, size-exclusion chromatography, immunocapture, sequencing, qPCR, and internal normalization strategies limits the comparability across studies [11,20,21]. Fourth, most clinical studies remain cross-sectional, small, and single-center, with heterogeneous definitions of diabetic complication, limited external validation, and insufficient longitudinal outcome assessment [39,66,71,105,118,119,120].

Based on the considerations stated above, future research must not be centered on describing novel miRNAs. Priority should be given to the standardization on protocols for vesicular characterization, robust miRNA quantification, clinical meaningful outcomes, and external validation [11,20,21]. For biomarker development, vesicular miRNA panels must demonstrate superior predictive value over conventional clinical tools. For therapeutic applications, the biodistribution, dosage, duration of effect, immunogenicity, tissue specificity, and risk of off-target effects must be clearly established [6,17,33,34,45,46,47]. Across these areas, integration with imaging, metabolomics, proteomics, and clinical data may accelerate the transition to precision medicine models.

Future integrated multi-organ studies should evaluate combined panels rather than isolated miRNAs. A clinically relevant design would include simultaneous assessment of circulating EV-miRNAs, organ-proximal samples such as urine for kidney injury and vitreous humor when clinically available for retinal disease, metabolic and inflammatory markers, liver imaging or fibrosis assessment, retinal imaging, neuropathy phenotyping, and longitudinal outcomes. Such studies would help determine whether shared EV-miRNA signatures represent systemic diabetic stress, organ-specific pathology, or true interorgan communication [66,67,68,69,70,71,105,118,119,120,131,132,133].

Overall, EV-associated miRNAs should not be interpreted solely as secondary markers of diabetic morbidity, but their role as functional mediators should be assigned only when supported by experimental transfer and target-engagement evidence. Current mechanistic and translational evidence supports a biologically plausible intercellular and interorgan communication model, while definitive human causal evidence remains limited. Their future value will depend on demonstrating whether these signals can be transformed from plausible biological associations into actionable clinical tools: reproducible biomarkers, stratification criteria, and, eventually, therapeutic vehicles or targets to prevent or mitigate multi-organ damage in diabetes. This integrative view is summarized in Figure 3 and complements the comparative synthesis presented in Table 1.

## 9. Limitations

This narrative review has several limitations. First, although the search strategy and synthesis were aligned with SANRA principles, this review was not designed as a systematic review and did not include formal risk-of-bias scoring, duplicate screening, protocol registration, or meta-analytic pooling [18]. Therefore, selection bias and citation bias cannot be excluded. Second, the literature on EV-associated miRNAs in diabetic complications is heterogeneous with respect to biological matrix, EV isolation or enrichment, vesicle characterization, RNA extraction, miRNA platform, normalization strategy and clinical outcome definition [11,19,20,21]. Third, many human studies measure total circulating miRNAs rather than EV-associated miRNAs and do not separate vesicular cargo from Argonaute-bound, lipoprotein-associated, or other non-vesicular extracellular RNA fractions [14,22,24]. Fourth, most clinical studies remain small, cross-sectional, and single-center, which limits causal inference, prognostic interpretation, and generalizability [39,66,71,94,105,118,119,120]. Fifth, most therapeutic and mechanistic evidence is derived from cell culture or animal models, with limited validation in human cohorts and no established EV-miRNA therapy for diabetic complications [33,34,45,46,47,48,49,117,135]. Finally, the proposed multi-organ model linking neuropathy, DKD, DR, and MASLD through EV-associated miRNAs should be considered a biologically plausible and hypothesis-generating framework rather than a proven human causal axis. Longitudinal, multicenter, externally validated, and methodologically standardized studies are required to determine whether EV-miRNA signatures can become clinically actionable biomarkers or therapeutic targets.

## 10. Conclusions

EV-associated miRNAs support a biologically plausible link between chronic hyperglycemia, metabolic inflammation, interorgan communication, and diabetic complications. Across neuropathy, DKD, DR, and MASLD, EV-associated miRNAs have been associated with shared processes such as oxidative stress, endothelial dysfunction, inflammation, fibrosis, angiogenesis, and tissue-specific injury. Although clinical implementation remains limited by methodological heterogeneity and insufficient longitudinal validation, this field offers a promising framework for biomarker discovery, risk stratification, and future targeted therapies.

At present, EV-associated miRNAs should be regarded as promising but investigational tools in diabetes care. Their potential value lies in their ability to integrate metabolic, inflammatory, vascular, neural, renal, retinal, and hepatic signals into measurable molecular signatures. However, clinical implementation will require rigorous distinction between EV-associated and total circulating miRNAs, standardized EV isolation and characterization, validated normalization strategies, adequately powered human cohorts, longitudinal outcome assessment, and external replication. Future progress will depend less on identifying additional isolated miRNAs and more on developing reproducible multi-miRNA panels linked to organ-specific sampling, clinical phenotyping, imaging, and meaningful longitudinal outcomes.

## Figures and Tables

**Table 1:** EV-associated and circulating miRNAs across diabetic complications: biological sources, proposed mechanisms, evidence level, biomarker potential, and translational limitations.

Complication/Axis	Sample Type/EV or Cellular Source	miRNA(s)	Proposed Mechanism	Evidence Level and Main Findings	Biomarker/Therapeutic Potential	Key Limitations	Ref.
Diabetic neuropathy: neuroinflammation and pain signaling	Dorsal root ganglion neurons, Schwann cells, microglia, neural/glial cells, neural endothelium; serum/plasma/whole blood in circulating-miRNA studies; EV/Exosomes when specifically isolated	miR-146a, miR-155, miR-9, miR-23a, miR-34a-5p	miR-146a downregulates IRAK1/TRAF6/NF-κB signaling; its reduction may amplify neuroinflammation. miR-155 and miR-9 participate in inflammatory and excitability-related pathways, whereas miR-34a-5p has been linked to painful diabetic neuropathy and microglial neuroinflammation.	Mainly preclinical and cross-sectional clinical evidence. Experimental restoration of miR-146a improves neuronal impairment in diabetic models. Peripheral blood miR-155/miR-146a and circulating miR-34a-5p have been evaluated as biomarker candidates, but most studies do not demonstrate EV-carrier specificity.	miR-146a replacement, miR-155 modulation, or pain-related miRNA panels could support anti-inflammatory strategies, painful-neuropathy subphenotyping, and treatment-response monitoring.	Discordant results due to matrix differences, small cohorts, cross sectional designs, heterogeneous neuropathy definitions, variable analytical platforms, and incomplete distinction between EV-associated and total circulating miRNAs.	[28,36,37,39,44]
Diabetic neuropathy: axonal injury, Schwann-cell dysfunction, and EV-mediated neural repair or injury	Schwann cells, sural nerve, dorsal root ganglia, Schwann cell-derived EVs/exosomes, MSC-derived EVs, adipose-derived stem cell EVs plasma exosomes	miR-21-5p, miR-34a, miR-140, Dicer dependent-miRNAs, miR-28, miR-31a, miR-130a, miR-130a-3p, miR-20b-3p	Schwann cell dysfunction impairs myelination, axonal repair, and regenerative programs. miR-21-5p is associated with axonal loss and axonal guidance/MAPK/Ras pathways. High-glucose-stimulated Schwann cell EVs may export pathogenic miRNA cargo, whereas reparative Schwann cell or stem cell EVs may support remyelination, autophagy regulation, and axonal recovery.	Human sural-nerve data show broad miRNA dysregulation and Schwann-cell-localized miR-21-5p associated with axonal loss. Preclinical EV studies show both pathogenic effects or high-glucose Schwann cell EVs and protective effects of Schwann cell-, MSC-, adipose stem cell-, or plasma-derived EVs on nerve conduction, myelination, autophagy, and nerve fiber density.	Therapeutic EVs enriched with protective miRNAs, including miR-146a, miR-130a-3p, or miR-20b-3p, may promote neural repair in preclinical models. Pathogenic EV cargo may also serve as a marker of Schwann-cell stress.	Predominantly preclinical evidence; limited human validation; incomplete standardization of EV source, purity, dose, route, biodistribution, target engagement, and long-term safety. EVs may be protective or harmful depending on donor-cell state.	[32,33,34,35,45,46,47,48,49]
Diabetic neuropathy: circulating biomarker panels and painful neuropathy	Serum, plasma, whole blood; circulating EVs only when isolated and characterized; most available clinical studies measure total circulating miRNAs	miR-148a-3p, miR-216a, miR-377, miR-128a, miR-155, miR-146a, miR-375, miR-34a-5p, miR-30c	Circulating profiles reflect neuroinflammation, axonal damage, oxidative stress, immune-cell composition, metabolic stress, and painful neuropathy phenotypes.	Cross-sectional human biomarker evidence. miR-148-3p showed high diagnostic performance in a discovery cohort, while miR-216a/miR-377 and miR-155/miR-146a combinations have been proposed as candidate panels. Whole-blood findings are partly discordant across studies.	Multi-miRNA panels could improve early detection, painful vs. painless neuropathy, risk stratification, and monitoring, but require longitudinal and external validation.	Small cohorts, cross-sectional designs, variable neuropathy definitions, inconsistent normalization, no meta-analytic EV-specific aggregation, and frequent lack of EV-carrier characterization.	[37,38,39,40,41,42,43,44]
Diabetic kidney disease: podocyte injury and EV-mediated glomerulotubular communication	Urinary EVs from podocytes, tubular epithelial cells, glomerular endothelium, and urinary tract cells; plasma EVs as systemic microvascular/inflammatory signals	miR-21, miR-192, miR-29, miR-30, miR-130a, miR-145, miR-155, miR-424, miR-136-5p	Hyperglycemia induces oxidative stress, AGEs, PKC activation, and TGF-β/Smad signaling, promoting podocyte loss, tubular apoptosis, extracellular matrix accumulation, and fibrosis. High-glucose-treated podocyte EVs can induce proximal tubular epithelial-cell apoptosis, supporting EV-mediated glomerulotubular injury propagation.	Preclinical mechanistic evidence plus human urinary EV biomarker studies. Urinary EV-miRNAs have been associated with albuminuria, eGFR, fibrosis-related pathways, and DKD risk. Urinary exosomal miR-136-5p has been proposed as a diagnostic marker and correlated with renal-risk indicators.	Urinary EV-miRNA panels may be evaluated for kidney-proximal early detection, risk stratification, and treatment monitoring before irreversible eGFR loss.	Urine collection, storage, proteinuria, hematuria, EV isolation, RNA normalization, and incomplete correlation with renal tissue or longitudinal outcomes limit comparability.	[50,51,52,64,65,66,67,68,69,92]
Diabetic Kidney disease: fibrosis, inflammation, endothelial dysfunction, and epitranscriptomic regulation	Podocytes, mesangial cells, proximal tubule, glomerular endothelium, urinary EVs, plasma/circulating miRNAs	miR-21, miR-29, miR-155, miR-214, miR-23b, miR-126, miR-221, miR-200 family, let-7c-5p, let-7b-5p, miR-29a-3p	miR-21 acts as a TGF-β-induced profibrotic node through Smad7/PTEN-related pathways. miR-29 family members counteract extracellular matrix accumulation. miR-146a/miR-155 regulate inflammation signaling, while miR-126 supports endothelial homeostasis.	Clinical and preclinical evidence. miR-21, miR-192, miR-29, miR-21-5p, and miR-30b-5p have been associated with DKD-related phenotypes. Longitudinal circulating miRNA evidence links TGF-β1-regulated miRNAs with rapid ESRD progression, although this evidence is not necessarily EV-specific.	Inhibition of miR-21, restoration of miR-29/miR-30, or modulation of endothelial miR-126 are candidate antifibrotic or vascular-repair strategies.	Many studies are not EV-specific; therapeutic strategies remain preclinical; miRNA pleiotropy, off-target effects, renal-compartment targeting, and lack of longitudinal EV validation remain major barriers.	[50,51,52,53,54,55,56,59,60,61,93]
Diabetic Retinopathy: early neurovascular damage and retinal EV communication	EVs from retinal endothelial cells, pericytes, Müller glia, microglia, retinal pigment epithelial cells, neural retina, serum/plasma, and vitreous humor.	miR-146a, miR-155, miR-21, miR-124, miR-26a-5p, miR-296-5p, miR-3976, miR-9-3p, miR-202-5p, miR-486-3p	DR combines microangiopathy, retinal neurodegeneration, glial activation, oxidative stress, inflammation, and blood–retinal barrier disruption. Müller glia-derived exosomal miR-9-3p may promote endothelial angiogenic behavior, whereas RPE-derived exosomal miR-202-5p may counteract high-glucose-induced EndoMT.	Mixed evidence from circulating biomarkers, vitreous studies, and preclinical functional EV models. Serum exosomal miR-3976 has been proposed as an early DR candidate. miR-146a acts as an anti-inflammatory brake, while miR-155 and miR-21 are linked to inflammation and barrier injury.	EV-miRNA panels may complement retinal imaging in future validation studies for early neurovascular stress. miR-486-3p-enriched MSC exosomes and modulation of inflammatory/glial miRNAs may complement anti-VEGF strategies in preclinical settings.	Vitreous humor is tissue-proximal but invasive and enriched for advanced disease. Serum/plasma are accessible but less retina-specific. Many studies are small, cross-sectional, and do not distinguish EV-miRNAs from total circulating miRNAs.	[94,95,96,97,98,99,100,101,102,103,106,112,113,114,117,119,120]
Diabetic retinopathy: angiogenesis, diabetic macular edema, and progression to proliferative disease	Retinal endothelium, pericytes, retinal pigment epithelium, Müller glia, vitreous humor, plasma/serum EVs, total circulating miRNAs	miR-126, miR-200b, miR-21, miR-181c, miR-1179, miR-142, miR-377-3p, miR-431-5p	Retinal hypoxia activates HIF-1/VEGF signaling and neovascularization. miR-126 supports endothelial homeostasis; miR-377-3p has been linked to VEGF regulation in diabetic macular edema; miR-431-5p has been proposed as a serum EV marker for proliferative DR.	Clinical biomarker evidence plus emerging EV-specific studies. Panels including miR-21, miR-181c, and miR-1179 have discriminated NPDR from PDR in selected cohorts. Serum exosomal miR-377-3p and serum EV-encapsulated miR-431-5p have been proposed as candidate markers. Registered human studies are evaluating exosome changes and plasma exosome proteomics in DR.	Serum/plasma and EV-miRNA panels may complement fundus photography, OCT/OCT-A, fluorescein angiography, and anti-VEGF response monitoring.	Small cohorts, cross-sectional designs, inconsistent DR staging, treatment exposure, macular edema heterogeneity, lack of external validation and incomplete separation of EV-associated vs. total circulating miRNAs.	[94,95,96,97,99,104,105,106,111,115,116,118,119,120,121,122]
Diabetes associated MASLD: liver–endothelium axis	EVs derived from steatotic/lipotoxic hepatocytes; vascular endothelium; circulating serum/plasma EVs	miR-1, miR-27a, miR-1297, miR-26a, miR-122-5p, miR-375-3p, miR-27b-3p, miR-30a-5p, miR-103a-3p, let-7d-5p, let-7f-5p	Hepatocyte lipotoxicity reprograms EV cargo. Palmitate-treated hepatocyte EVs can transfer miR-1 to endothelial cells, activate NF-κβ, reduce KLF4, and promote vascular inflammation and atherogenesis. Human EV miRNome data also link EV-miRNAs to lipid metabolism, inflammation, steatohepatitis, and significant fibrosis.	Strong preclinical mechanistic evidence for hepatocyte-to-endothelium EV transfer; emerging human EV-miRNA biomarker evidence in biopsy-proven MASLD and serum EV cohorts.	AntagomiR-1, reduction in hepatocyte EV inflammatory cargo, and EV-miRNA panels may help stratify hepatic and vascular risk if externally validated, but clinical therapeutic validation is lacking.	Human liver-derived EV specificity is difficult to establish in serum/plasma without donor-cell markers. Direct causal evidence linking MASLD EV-miRNAs to diabetic microvascular complications in humans remain limited.	[126,128,131,133]
Diabetes associated MASLD: liver-pancreas β-cell axis and metabolic progression	EVs derived from steatotic hepatocytes; pancreatic β cells as recipient cells; macrophage-derived exosomes in obesity-related cardiometabolic inflammation	miR-126a-3p	Steatotic hepatocyte EVs can promote pancreatic β-cell apoptosis and worsen diabetes via miR-126a-3p targeting IRS-2, supporting a liver-β-cell communication model.	Preclinical evidence suggests that MASLD may influence diabetes progression beyond being a hepatic comorbidity. Macrophage exosome studies also support EV-mediated cardiometabolic inflammation in obesity.	Anti-miR strategies or modification of hepatic EV cargo could theoretically protect β-cells function in selected scenarios.	Human β-cell physiology is heterogeneous; longitudinal human validation, EV-carrier specificity, donor-cell attribution, and safety data are lacking.	[129,134]
MASLD associated with diabetes: inflammation, fibrosis, and EV-miRNA biomarkers panels	Liver tissue, hepatocyte-derived EVs serum/plasma EVs, total serum/plasma miRNAs, vesicular and non-vesicular extracellular RNA carriers	miR-21, miR-34a, miR-122, miR-99a, miR-141-3p, miR-192-5p, miR-574-3p, miR-542-3p, miR-200a-3p, miR-27b-3p, miR-30a-5p, miR-103a-3p, let-7d-5p, let-7f-5p	miR-21, miR-34a, and miR-122 are associated with inflammation, redox stress, and fibrosis. miR-99a has been linked to MASLD in T2D, HbA1c, IL-6, and mTOR. EV-miRNA profiles may capture lipid dysregulation, chronic inflammation, liver injury, steatohepatitis, and significant fibrosis.	Human biomarker evidence, including emerging EV-specific MASLD studies. Serum EV-associated miR-574-3p, miR-542-3p, and miR-200a-3p have been proposed as a diagnostic panel. EV versus total serum comparisons suggest that vesicular and non-vesicular miRNA compartments may provide non-equivalent information.	Integrated EV-miRNA panels may complement liver enzymes, fibrosis scores, elastography, imaging, glycemic control, and inflammatory markers. Metabolic therapies may modulate miRNA networks, but EV-specific therapeutic implications remain investigational.	Carrier identity is frequently undefined; serum/plasma EVs may reflect liver, adipose tissue, platelet, endothelial, immune, renal, or systemic metabolic signals. Panels require external validation, fibrosis-stage adjustment, and longitudinal outcomes.	[123,125,130,131,132,133,136,138]
MASLD as a systemic amplifier and experimental therapeutic EV node	MSC-derived EVs, liver-targeted EVs, hepatic macrophages, hepatocytes, cerebral microvasculature, systemic circulating EVs.	miR-31-5p, miR-483-5p, miR-5120, miR-182-5p	MASLD may function as a systemic communication node. MSC-derived EVs can accumulate in the liver, deliver miR-31-5p, modulate PDGFB in hepatic macrophages, and improve hepatic and neurovascular phenotypes in preclinical T2D/MASLD models.	Preclinical evidence supports the concept that modifying the hepatic EV environment may influence extrahepatic phenotypes. miR-483-5p is a candidate shared node across T2D, fatty liver, nephropathy, and neurological injury, but this remains hypothesis-generating.	Therapeutic EVs, miRNA mimics/inhibitors, weight loss, and antidiabetic drugs may modify hepatic EV cargo in experimental or selected clinical settings, but this remains investigational.	Direct causal evidence linking MASLD, neuropathy, DKD, and DR through EV-miRNA transfer in the same human cohorts is still lacking. Therapeutic translation requires donor-cell selection, EV purity, dose, biodistribution, target engagement, and long-term safety.	[125,133,135,137,139,140,141]

Note: Evidence level refers to the dominant evidence supporting each axis. “Biomarker level” indicates association with disease status, stage, or outcome without proof of causality. “Functional preclinical EV evidence” indicates experimental support for vesicle uptake, cargo transfer, target engagement, or phenotypic effects in cell or animal models. Clinical associations based on serum/plasma total miRNAs should not be interpreted as EV-mediated unless EV isolation and characterization were performed. Abbreviations: DKD, diabetic kidney disease; DN, diabetic neuropathy; DR, diabetic retinopathy; EVs, extracellular vesicles; MASLD, metabolic dysfunction-associated steatotic liver disease; MSCs, mesenchymal stromal/stem cells; NPDR, non-proliferative diabetic retinopathy; PDR, proliferative diabetic retinopathy. When vesicle biogenesis was not demonstrated, “EVs” or “EV-enriched preparations” should be interpreted as operational terminology rather than definitive evidence of exosome or microvesicle origin.

## Data Availability

No new data were created or analyzed in this study. Data sharing is not applicable to this article.

## Supplementary Materials

The following supporting information can be downloaded at: https://www.mdpi.com/article/10.3390/metabo16070500/s1, Table S1: Methodological stratification of EV-associated and circulating miRNA evidence across diabetic complications.

## Abbreviations

The following abbreviations are used in this manuscript:

AGEs	Advanced glycation end products
Ago2	Argonaute 2
ALIX	ALG-2-interacting protein X
ANGPTL4	Angiopoietin-like 4
AUC	Area under the curve
CTGF	Connective tissue growth factor
DKD	Diabetic kidney disease
DN	Diabetic neuropathy
DR	Diabetic retinopathy
eGFR	Estimated glomerular filtration rate
ESCRT	Endosomal sorting complex required for transport
EVs	Extracellular vesicles
GAP-43	Growth-associated protein 43
HbA1c	Glycated hemoglobin
HIF-1	Hypoxia-inducible factor 1
IL-6	Interleukin 6
IRAK1	Interleukin-1 receptor-associated kinase 1
IRS-2	Insulin receptor substrate 2
JAK/STAT	Janus kinase/signal transducer and activator of transcription
MAPK	Mitogen-activated protein kinase
MASLD	Metabolic dysfunction-associated steatotic liver disease
MASH	Metabolic dysfunction-associated steatohepatitis
MSCs	Mesenchymal stromal cells
miRNAs	microRNAs
MVBs	Multivesicular bodies
NAFLD	Non-alcoholic fatty liver disease
NF-κβ	Nuclear factor kappa B
Nox4	NADPH oxidase 4
OCT	Optical coherence tomography
PDGFB	Platelet-derived growth factor subunit B
PI3K/Akt	Phosphoinositide 3-kinase/protein kinase B
PKC	Protein kinase C
PLD1	Phospholipase D1
PPAR-α	Peroxisome proliferator-activated receptor alpha
PTEN	Phosphatase and tensin homolog
qPCR	Quantitative polymerase chain reaction
RAGE	Receptor for advanced glycation end products
ROC	Receiver operating characteristic
ROS	Reactive oxygen species
SANRA	Scale for the Assessment of Narrative Review Articles
SC-Exo	Schwann cell-derived exosomes
Smad	Small mothers against decapentaplegic
SOCS1	Suppressor of cytokine signaling 1
SOX10	SRY-box transcription factor 10
T2D	Type 2 diabetes
TGF-β	Transforming growth factor beta
TRAF6	TNF receptor-associated factor 6
TSG101	Tumor susceptibility gene 101
VEGF	Vascular endothelial growth factor
VEGF-A	Vascular endothelial growth factor A
YBX1	Y-box binding protein 1
