# Supplementary material for: Extracellular Vesicle-Associated microRNAs as Candidate Biomarkers and Mediators of Diabetic Complications: Clinical and Translational Evidence Across Neuropathy, Diabetic Kidney Disease, Retinopathy, and MASLD
Source: Metabolites. 2026 Jul 16;16(7):500. doi: 10.3390/metabo16070500 (PMC13413715; doi:10.3390/metabo16070500)
Supplement: Supplementary file 1 [file metabolites-16-00500-s001.zip › metabolites-4385381-supplementary.pdf]

Supplementary material.

Extracellular Vesicle-Associated microRNAs as Candidate Biomarkers and Mediators of Diabetic Complications: Clinical and Translational Evidence Across Neuropathy, Diabetic Kidney Disease, Retinopathy, and MASLD.

Supplementary Table S1. Methodological stratification of EV-associated and circulating miRNA evidence across diabetic complications

Complication / axis	Representative study or evidence group	Species / population	Sample type	EV-associated vs total miRNA	EV isolation / enrichment	EV characterization / reporting	miRNA analysis / normalization	Study design	Sample size / validation	Outcome definition	Evidence level	Main limitation	Ref.
**Diabetic neuropathy: neuroinflammation**	miR-146a in dorsal root ganglia	Experimental diabetic neuropathy	Dorsal root ganglia	Cellular/tissue miRNA, not EV-specific	Not applicable; not an EV study	Not applicable; tissue /cellular miRNA study	TaqMan miRNA RT-qPCR; relative quantities calculated using 2-ΔΔCt method; U6 snRNA TaqMan miRNA control assay used as endogenous control; calibrated to wild-type samples	Preclinical mechanistic	Animal model	Neuronal impairment, inflammation	Mechanistic	Not EV-specific; human validation needed	[28]
**Diabetic neuropathy: oxidative stress**	miR-25 and miR-106a models	Experimental diabetic neuropathy	Neural tissue / DRG	Cellular/tissue miRNA, not EV-specific	Not applicable; not an EV study	Not applicable; tissue / cellular miRNA study	qPCR; GAPDH mRNA or U6 snRNA used to normalize relative expression levels of target mRNAs/miRNAs, as reported [29]; NR in available files	Preclinical mechanistic	Animal model	ROS, Nox4, AGE-RAGE, 12/15-LOX	Mechanistic	Not EV-specific; model-dependent	[29,30]
**Diabetic neuropathy: Schwann-cell injury**	Human sural nerve miRNA profiling	Humans with DN	Sural nerve	Tissue miRNA, not EV-specific	Not applicable; not an EV study	Not applicable; human nerve tissue study	Small RNA sequencing with integrated miRNA—mRNA analysis; bioinformatic normalization as reported	Observational tissue study	Limited human tissue availability	Axonal loss, Schwann-cell localization	Human tissue association	Preprint / observational; no EV transfer	[35]
**Diabetic neuropathy: Schwann-cell EV therapy**	Schwann cell-derived exosomes	Diabetic mice / Schwann-cell models	Cell culture supernatant; experimental EVs	EV-associated	Differential ultracentrifugation from Schwann-cell conditioned medium	Exosome characterization reported: particle size / number by NanoSight / NTA, TEM, and exosomal markers. Reported markers include Alix, CD9, CD81 and calnexin in [33], and Alix, CD63 and HSP70 in [34]	qRT-PCR/TaqMan miRNA assays with functional testing; U6 or endogenous control as reported	Preclinical interventional	Animal models	Nerve conduction, myelination, axonal growth	Functional preclinical EV evidence	Dose, purity, biodistribution, and safety not validated in humans	[33,34]
**Diabetic neuropathy: MSC-EV therapy**	MSC-derived exosomes and engineered miR-146a EVs	Diabetic mice	MSC-conditioned medium / EVs	EV-associated	Ultracentrifugation from MSC-conditioned medium	Reported by original studies: TEM, NanoSight / NTA particle analysis, and Western blot for exosomal markers	qPCR / miRNA functional assays; U6 snRNA as endogenous control	Preclinical interventional	Animal models	Nerve conduction, sensitivity, nerve fiber density	Functional preclinical EV evidence	Human translation, manufacturing, and long-term safety unknown	[45,46]
**Diabetic neuropathy: pathogenic Schwann-cell EVs**	High-glucose-stimulated Schwann cell exosomes	Cell and mouse models	Schwann-cell conditioned medium	EV-associated	Differential ultracentrifugation from high-glucose Schwann-cell conditioned medium	Reported: exosomal marker proteins Alix, CD63 and HSP70; particle number / size by NanoSight; functional uptake / localization assays	qRT-PCR / TaqMan miRNA assays; U6 reference miRNA; gain / loss-of-function and functional assays	Preclinical mechanistic	Animal / cell models	Axonal growth, nerve conduction, intraepidermal nerve fibers	Functional preclinical EV evidence	Highlights EVs can be harmful depending on donor-cell state	[32]
**Diabetic neuropathy: stem cell EV-miRNAs**	Adipose-derived stem cell EV miR-130a-3p	Cell and animal models	Stem-cell EVs	EV-associated	Ultracentrifugation-based EV isolation from adipose-derived stem-cell conditioned medium	Reported by original study: EV morphology / size and marker assessment; details vary by assay	qRT-PCR; relative expression calculated using 2-ΔΔCt; GAPDH used for mRNA, U6 used for cellular miRNA, and cel-miR-39 used for EV miRNA	Preclinical interventional	Animal / cell models	Schwann-cell apoptosis, proliferation, nerve function	Functional preclinical EV evidence	Human translation unavailable	[48]
**Diabetic neuropathy: plasma exosomes**	Plasma exosomal miR-20b-3p	Type 1 diabetic rats	Plasma exosomes	EV-associated	Ultracentrifugation from rat plasma	Reported by original study: EV / exosome morphology, particle analysis and exosomal markers	qPCR / miRNA profiling with functional validation; relative expression calculated using 2-ΔΔCt	Preclinical interventional	Animal model	Schwann-cell autophagy, neuropathy phenotype	Functional preclinical EV evidence	Animal evidence only	[49]

Supplementary material.

Extracellular Vesicle-Associated microRNAs as Candidate Biomarkers and Mediators of Diabetic Complications: Clinical and Translational Evidence Across Neuropathy, Diabetic Kidney Disease, Retinopathy, and MASLD.

**Diabetic neuropathy: circulating biomarkers**	miR-148a-3p, miR-216a, miR-377, miR-155, miR-146a	Human T2D with/without DN	Serum, plasma, whole blood	Mostly total circulating miRNAs	Not EV-specific in most studies; no vesicle isolation in circulating biomarker studies	Vesicular carrier not characterized in most studies	qPCR / targeted circulating miRNA assays; normalization heterogeneous or not consistently reported across studies.	Cross-sectional / case-control	Mostly small cohorts; limited validation	DN diagnosis, pain, metabolic variables, ROC curves	Biomarker-level	Small samples, variable DN definitions, heterogeneous normalization, no EV-carrier distinction	[37–44]
**DKD: urinary EV diagnostics**	Selected urinary EV-miRNAs	Human DKD cohorts	Urine	EV-associated	Urinary EV isolation; approach varies across discovery, replication and external datasets	Extensive uEV reporting in the primary study, including NTA, TEM, immunodetection of CD9 / CD81 / CD63, ExoView / SP-IRIS and RNA quality assessment	Small RNA sequencing plus qPCR validation; normalization and differential expression analysis as reported, including DESeq2 / normalized count-based approaches and qPCR reference-miRNA validation	Multi-cohort biomarker study	Includes replication cohorts	Early DKD diagnosis	Biomarker-level EV evidence	Requires broader longitudinal validation	[52]
**DKD: podocyte–tubule EV communication**	High-glucose podocyte EVs	Cell models	Podocyte-derived EVs	EV-associated	ExoQuick-TC precipitation from high-glucose podocyte-conditioned medium	Reported by original study; NTA / NanoSight, TEM / electron microscopy, Western blot markers including CD63, CD9, Alix, and negative marker assessment	Primarily functional EV-transfer assays; miRNA quantification not the central analytical platform	Preclinical mechanistic	Cell model	Proximal tubular epithelial-cell apoptosis	Functional preclinical EV evidence	Cell model; human validation lacking	[65]
**DKD: urinary exosomal miRNAs**	miR-21-5p, miR-30b-5p	Human DKD, CKD and T2D alone	Urine	EV-associated / exosomal	miRCURY Exosome Isolation Kit from urine supernatant	Limited exosome characterization in the biomarker study; focused mainly on urinary exosomal RNA rather than full EV subtype validation	miRCURY LNA miRNA urine focus PCR panels and validation PCR assays; UniSp3 inter-plate calibrator, UniSp6 spike-in, and NormFinder-selected stable reference miRNAs	Cross-sectional biomarker	Limited cohorts	Early DKD diagnosis / monitoring	Biomarker-level	Variable urine processing and normalization	[68]
**DKD: incipient nephropathy**	miR-130a, miR-145, miR-155, miR-424	Human Type 1 diabetes with / without microalbuminuria	Urinary exosomes	EV-associated / exosomal	Differential centrifugation, filtration, and two consecutive ultracentrifugation steps from urine	Reported: NanoSight particle analysis; Western blot for Hsp70, Alix and calnexin; selected RNase treatment; exosomal pellet characterization	TaqMan miRNA array and TaqMan qPCR; U6 snRNA used for standardization	Cross-sectional biomarker	Limited cohort	Incipient nephropathy / microalbuminuria	Biomarker-level	Small sample; no longitudinal hard renal outcomes	[69]
**DKD: circulating EV origin**	EV-miRNA profiles across DKD stages	Human DKD	Plasma / circulating EVs	EV-associated	Circulating EV isolation; cell-origin profiling by flow cytometry / EV phenotyping approach	EV profiling reported by original study; focus on cellular origin and EV-miRNA profiles rather than organ-specific renal EV validation	miRNA profiling / qPCR as reported; normalization heterogeneous and plasma EVs are systemic	Cross-sectional	Limited cohort	DKD stage	Biomarker-level	Plasma EVs are systemic and not kidney-specific	[70]
**DKD: urinary exosomal miR-136-5p**	miR-136-5p diagnostic study	Human T2D with/without DKD	Urinary exosomes	EV-associated / exosomal	Sequential centrifugation and ultracentrifugation from urine; final exosome pellet stored at -80°C	Reported: urinary exosome morphology by electron microscopy / TEM, particle-size assessment, and Western blot markers including CD63, TSG101 and negative marker calnexin	RT-qPCR validation of miR-136-5p; relative expression calculated using 2-ΔΔCt; Caenorhabditis elegans miR-39-3p used as external reference control	Cross-sectional biomarker	Single study; needs external validation	DKD diagnosis, UACR, eGFR-related indicators	Biomarker-level	Small cohort, no longitudinal renal outcomes	[92]
**DKD: longitudinal circulating miRNAs**	TGF-β1-regulated circulating miRNAs	Human Type 1 diabetes	Plasma / circulating	Total circulating miRNAs, not necessarily EV-specific	Not EV-specific; circulating miRNA study	Not applicable; vesicular carrier not characterized	Circulating miRNA profiling / RT-qPCR; spike-in and normalization strategy as reported	Longitudinal cohort	Long follow-up	Rapid ESRD progression	Prognostic biomarker evidence	Valuable longitudinal design but not EV-specific	[93]

Supplementary material.

Extracellular Vesicle-Associated microRNAs as Candidate Biomarkers and Mediators of Diabetic Complications: Clinical and Translational Evidence Across Neuropathy, Diabetic Kidney Disease, Retinopathy, and MASLD.

**DR: circulating miRNA meta-evidence**	Circulating miRNAs for DR stage	Human DR	Serum/plasma	Mostly total circulating miRNAs	Not EV-specific; evidence synthesized from circulating miRNA studies	Not applicable; EV carrier generally not characterized across included studies	Platforms and normalization heterogeneous across included studies; not extractable at evidence-group level	Systematic review / meta-analysis	Multiple studies	DR stage identification	Biomarker-level	Not EV-specific; heterogeneous platforms	[94]
**DR: vitreous miRNAs**	Vitreous miRNA profiling	Human PDR / retinal disease	Vitreous humor	Total vitreous miRNAs; EV status variable	Mixed; vitreous miRNA studies and review-level evidence, not uniformly EV-specific	Heterogeneous across included studies; vitreous is tissue-proximal but EV subtype reporting varies	miRNA profiling / qPCR / sequencing depending on study; normalization heterogeneous or review-level	Observational / systematic evidence	Surgical cohorts	PDR, anti-VEGF exposure, retinal disease	Tissue-proximal biomarker	Invasive; enriched for advanced disease	[119,120]
**DR: retinal inflammation**	miR-146a, miR-155, miR-21, miR-124	Retina / experimental systems	Retinal tissue, serum/plasma , vitreous	Often total miRNA	Mostly not EV-specific; retinal tissue / cell and circulating evidence	Not applicable or heterogeneous; EV carrier often not characterized	qPCR / experimental assays / review-level synthesis; normalization heterogeneous	Preclinical + clinical associations	Heterogeneous	Inflammation, barrier disruption, angiogenesis	Mechanistic + biomarker	EV-carrier status often missing	[100–103]
**DR: serum exosomal miR-3976**	Early DR exosomal candidate	Human early DR + cell assays	Serum exosomes	EV-associated / exosomal	Differential ultracentrifugation from serum	Reported: TEM, NTA / ZetaView particle analysis, Western blot markers including CD63, CD9 and TSG101, and exosome uptake assays	Small RNA sequencing / miRNA profiling plus qRT-PCR validation; U6 used to normalize miRNA expression and 18S used to normalize mRNA expression	Small clinical + functional study	Small cohort	Early DR, retinal cell stress	EV biomarker + exploratory functional	Small sample; external validation needed	[114]
**DR: diabetic macular edema**	Serum exosomal miR-377-3p	Human T2D with/without DME	Serum exosomes	EV-associated / exosomal	ExoQuick precipitation from serum	Reported: TEM / electron microscopy, NTA, and Western blot markers including CD63 and CD9	RT-qPCR and functional assays in retinal pigment epithelial cells; GAPDH as internal control and 2-ΔΔCt for analysis; miRNA microarray data normalized using the 75% quartile method	Cross-sectional + in vitro	Limited cohort	Diabetic macular edema, VEGF regulation	EV biomarker + mechanistic	Needs larger validation and adjustment for treatment exposure	[115]
**DR: proliferative DR EV biomarker**	Plasma-derived sEV miR-431-5p	Human PDR	Plasma-derived sEVs	EV-associated	Plasma sEV isolation by sequential differential centrifugation and ultracentrifugation.	TEM, NanoSight / NTA size distribution, and Western blot for CD9 and CD63	Agilent miRNA microarray with quantile normalization; RT-PCR validation. U6 was used for sEV miRNAs, cel-miR-39 spike-in for plasma miRNAs, and GAPDH for mRNA targets	Cross-sectional biomarker	Limited cohort	PDR	EV biomarker-level	Small discovery / validation design; EV biomarker-level evidence; no longitudinal progression data	[116]
**DR: Müller glia EV transfer**	Exosomal miR-9-3p	Cell / vitreous-derived evidence	Müller glia / vitreous exosomes	EV-associated / exosomal	Exosome isolation from vitreous humor and Müller glia conditioned medium	Reported: NTA, TEM / electron microscopy, Western blot markers including CD63, CD81, TSG101, and negative marker calnexin; uptake / functional assays	Small RNA sequencing / microarray-informed analysis plus qRT-PCR; U6 or endogenous normalization as reported; functional validation S1P1 / VEGFR2 axis	Preclinical mechanistic	Cell and experimental models	Angiogenesis via S1P1/VEGFR2	Functional EV evidence	Human causal validation limited	[112]
**DR: RPE protective EVs**	Exosomal miR-202-5p	Retinal pigment epithelial cell models	RPE-derived exosomes	EV-associated / exosomal	ARPE-19 conditioned-medium exosome isolation by sequential centrifugation, filtration, and ultracentrifugation	TEM showing 50—150 nm vesicles, Western blot for CD63 and CD9, and PKH67 uptake by HUVECs	qPCR / functional assays of miR-202-5p and the TGFβR2 / TGFβ / Smad pathway; U6 used as miRNA internal control	Preclinical mechanistic	Cell models	EndoMT, TGFβR2/TGFβ/Smad	Functional EV evidence	Cell-model evidence; uses ARPE-19 / HUVEC system; therapeutic delivery and human safety remains unknown	[113]
**DR: MSC-EV therapy**	Exosomal miR-486-3p	Preclinical DR models	Bone marrow MSC exosomes	EV-associated / exosomal	BMSC-derived exosome isolation using exosome-free	TEM morphology, flow cytometry for CD63 / CD81, Western blot for	qPCR / functional assays of miR 486-3p and TLR4 / NF-	Preclinical interventional	Animal / cell models	TLR4/NF-κB repression,	Therapeutic preclinical	No human safety or efficacy data	[117]

Supplementary material.

Extracellular Vesicle-Associated microRNAs as Candidate Biomarkers and Mediators of Diabetic Complications: Clinical and Translational Evidence Across Neuropathy, Diabetic Kidney Disease, Retinopathy, and MASLD.

					serum, filtration, ultrafiltration, and ExoQuick-TC precipitation	CD9 / CD63, and PKH26 uptake by Müller cells	κβ axis; U6 for miR-486-3p, β-actin for mRNAs, and 2-ΔΔCt quantification			retinal protection			
**MASLD: hepatocyte–endothelium axis**	Hepatocyte EV miR-1	Experimental MASLD/lipotoxicity	Hepatocyte-derived EVs	EV-associated	Differential ultracentrifugation from palmitate-treated hepatocyte-conditioned medium	Reported: NTA / NanoSight particle analysis, TEM / electron microscopy, Western blot markers including CD63, CD81 and TSG101, and uptake assays	qPCR / functional assays; U6 small RNA and cel-miR-39 used as internal and external standards; miR-1 inhibition / antagomiR functional validation	Preclinical mechanistic	Cell / animal models	Endothelial inflammation, atherogenesis	Functional EV evidence	Acute lipotoxicity models; human validation limited	[128]
**MASLD: liver–β-cell axis**	Steatotic hepatocyte EV miR-126a-3p	Experimental MASLD/T2D	Hepatocyte-derived EVs	EV-associated	Differential ultracentrifugation from steatotic hepatocyte-conditioned medium and serum-derived EVs in mouse models	Reported: NTA particle concentration / size and EV uptake assays; EV characterization as described in original study	qRT-PCR / functional assays; U6 used for internal control and cel-miR-39 as external reference; IRS-2 target validation	Preclinical mechanistic	Cell / animal models	β-cell apoptosis, IRS-2 targeting	Functional EV evidence	Human β-cell translation uncertain	[129]
**MASLD: macrophage exosomes**	IL-4 polarized macrophage exosomes	Obesity / diabetes models	Macrophage exosomes	EV-associated / exosomal	Macrophage-derived exosome isolation as reported by original study	Reported by original study; focus on macrophage exosome-mediated cardiometabolic inflammation rather than MASLD-specific liver EVs	Functional assays; not primarily a miRNA quantification study	Preclinical mechanistic	Animal / cell models	Cardiometabolic inflammation	Functional EV evidence	Not MASLD-specific; human validation lacking	[134]
**MASLD: circulating miRNAs**	miR-21, miR-34a, miR-122, miR-99a	Human MASLD with/without T2D	Serum/plasma	Mostly total circulating miRNAs	Not EV-specific; serum/plasma total miRNA evidence	Not applicable; vesicular carrier not defined	qPCR / targeted circulating miRNA assays; normalization as reported; carrier identify not established.	Cross-sectional clinical	Human cohorts	MASLD, redox status, inflammation, HbA1c, IL-6, mTOR	Biomarker-level	Carrier not defined; confounding by obesity/T2D	[123,130]
**MASLD: EV miRNome**	EV miRNome profiling in MASLD	Human MASLD	Circulating EVs	EV-associated	Serum EV isolation using ExoQuick precipitation	EV characterization reported by original study; includes EV marker / particle reporting as available	EV miRNome profiling; RNA extraction with miRNeasy serum / plasma approach; normalization and bioinformatic analysis as reported	Clinical biomarker	Human cohort	Steatohepatitis, significant fibrosis, at-risk MASH	EV biomarker-level	Needs external validation and longitudinal outcomes	[131]
**MASLD: serum EV panel**	EV miR-574-3p, miR-542-3p, miR-200a-3p	Human MASLD	Serum EVs	EV-associated	Serum EV isolation as reported by original study	Reported: EV/exosome characterization in original study; details include particle and/or marker assessment as available	miRNA profiling and qPCR validation of miR-574-3p, miR-542-3p and miR-200a-3p; normalization as reported	Clinical biomarker	Human cohort	MASLD diagnosis	EV biomarker-level	Requires external validation and fibrosis-stage adjustment	[132]
**MASLD: EV vs total serum miRNA**	EV characteristics and miRNA transport across MASLD	Human MASLD spectrum	Serum EVs and total serum	EV-associated vs total serum miRNAs	Serum EV isolation and comparison with total serum miRNA compartment	EV characteristics reported; explicitly compares EV-associated and total serum miRNA compartments	miRNA assays in EVs and serum; normalization as reported; useful for carrier-specific interpretation rather than causal inference	Clinical biomarker	Human cohort	MASLD spectrum	EV vs non-EV compartment evidence	Need longitudinal outcome validation	[133]
**MASLD: MSC-EV therapy**	UC-MSC EV miR-31-5p	T2D/MASLD model	MSC-derived EVs	EV-associated	Umbilical cord MSC-derived EV isolation as reported by original study	Reported by original study; therapeutic EV model with biodistribution / liver accumulation assessment	qPCR / functional assays for miR-31-5p / PDGFB axis; normalization as reported	Preclinical interventional	Animal model	Liver phenotype, hepatic macrophages, neurovascular health	Therapeutic preclinical	Human dose, biodistribution, safety, and target engagement unknown	[135]

Supplementary material.

Extracellular Vesicle-Associated microRNAs as Candidate Biomarkers and Mediators of Diabetic Complications: Clinical and Translational Evidence Across Neuropathy, Diabetic Kidney Disease, Retinopathy, and MASLD.

**MASLD: shared miRNA nodes**	miR-483-5p, miR-5120, miR-182-5p	Human / experimental literature	Liver tissue, circulation, experimental models	Mixed; often not EV-specific	Mixed evidence; often not EV-specific	Heterogeneous or not extractable at evidence-group level	Variable across review / mechanistic / biomarker studies.	Review / mechanistic / biomarker evidence	Heterogeneous	T2D, fatty liver, nephropathy, neurological injury, hepatic steatosis	Hypothesis-generating	Shared miRNA does not prove EV-mediated multi-organ causality	[137,139–141]

Note: For rows summarizing more than one study, methodological fields indicate the dominant or reported approach when extractable from the primary articles. When the row represents a review, meta-analysis, registered study, evidence group, or mixed clinical literature, EV characterization and miRNA normalization are reported as heterogeneous, not consistently reported, or not extractable at evidence-group level. "As reported by original study" indicates that the primary article reported the method, but this table does not reproduce all technical details. Primary studies should be consulted for complete centrifugation steps, EV markers, particle analysis, RNA extraction kits, spike-ins, endogenous normalizers, and statistical workflows.

Abbreviations: N/A, not applicable, NR, not reported or not clearly reported; EV, extracellular vesicle; qPCR, quantitative polymerase chain reaction; NTA, nanoparticle tracking analysis; TEM, transmission electron microscopy.
